# Autophagy in doxorubicin resistance: basic concepts, therapeutic perspectives and clinical translation

**DOI:** 10.3389/fimmu.2025.1642050

**Published:** 2025-09-30

**Authors:** Yantao Zhang, Yanqin Ji, Yanyang Tu, Yi Li

**Affiliations:** ^1^ Department of Clinical Medicine, The Fifth Clinical Institute, Zunyi Medical University, Zhuhai, Guangdong, China; ^2^ Department of Gynaecology, Huizhou Central People’s Hospital, Guangdong Medical University, Huizhou, Guangdong, China; ^3^ Huizhou Central People’s Hospital Academy of Medical Sciences, Huizhou, Guangdong, China; ^4^ Science Research Center, Huizhou Central People’s Hospital, Huizhou, Guangdong, China; ^5^ Science Research Center, Huizhou Central People’s Hospital, Guangdong Medical University, Huizhou, Guangdong, China; ^6^ Neurosurgery Department, The Second Affiliated Hospital of Zunyi Medical University, Xinlong Avenue and Xinpu Avenue Interchange, Zunyi, Guizhou, China

**Keywords:** autophagy, drug resistance, doxorubicin, cancer therapy, clinical translation

## Abstract

Doxorubicin (DOX) is still one of the leading compounds for cancer chemotherapy, but its clinical application has been restricted by the drug resistance. The emerging evidence has demonstrated that autophagy is a meticulously regulated by the lysosomal degradation as a regulator of this drug resistance. Autophagy can exert a pro-survival strategy under therapeutic stress through recycling cellular components, inhibiting apoptosis and remodelling metabolism, thereby enhancing carcinogenesis. The present review aims to highlight the interaction between autophagy and DOX resistance, providing the molecular machinery of autophagy and its control by genetic factors, microenvironmental factors and non-coding RNAs. Mechanistically, autophagy can be considered as protective or cytotoxic, relying on the cellular context, but in most cases, autophagy serves as a survival pathway promoting chemoresistance. The present review will also discuss about the function of DOX in autophagy induction through ROS generation, DNA damage response and AMPK/mTOR axis, whereas providing context-specific adaptations including mitophagy in cancer stem cells and lysosomal remodelling. The pre-clinical studies have highlighted the function of pharmacological compounds and nanoparticles for the regulation of autophagy for improving DOX sensitivity in cancer, accelerating therapeutic index. The strategies have focused on the application of small-molecule inhibitors, natural compounds, nanocarrier-mediated co-delivery of DOX with autophagy modulators and the development of combination therapeites providing the crosstalk of autophagy and cell death mechanisms in DOX resistance. The clinical translation depends on the development of more effective autophagy-targeted drugs in combination therapies. Hence, the present review highlights the role of autophagy as a biomarker and therapeutic factors in reversing DOX resistance. By elucidating the complex biology linking autophagy to drug resistance, it is emphasized that tailored approaches integrating autophagy modulation may yield more effective and less toxic cancer treatments.

## Highlights

- Autophagy enhances tumor survival by degrading cellular components, facilitating resistance to doxorubicin, a versatile anticancer drug.
- Targeting autophagy pathways presents a promising strategy to overcome doxorubicin resistance in cancer treatment.
- Crosstalk between autophagy, apoptosis, and ferroptosis underlines complex mechanisms of drug resistance and cell death.
- Preclinical studies suggest autophagy modulation can reverse doxorubicin resistance, improving therapeutic outcomes.
- The development of molecularly targeted autophagy-modulating drugs could offer new, less toxic cancer therapies.

## 1 Introduction

In the mid-19th century, Rudolf Virchow’s “cellular theory” concluded that all diseases, including cancer are a result of alterations in cells, resulting in an understanding of cancer as a disease of abnormal cell proliferation (1). The cancer cells are considered as proliferating cells generating tumors with enhanced growth and metastasis (2, 3). Moreover, the recent advances in molecular biology has manifested a cascade in which driver mutations occur and upon activation, the tumor cells proliferate in an uncontrolled way lacking differentiation and increased invasion to the healthy tissues (4, 5). The conceptualization of cancer is considered as “a disease of the genes,” with mutations mainly regarded as drivers and selective forces in a dynamically changing population and microenvironment. It should be mentioned that a main characteristic of evolution is variations in the frequency of genes within a population. However, in case of cancer, it is beyond mere somatic evolution or a genetically heterogeneous cell population. Cancer cells evolve adaptations to increase their uptake of resources, co-opt normal cells, evade the immune system, and tolerate acidic conditions (6, 7). Therefore, cancer cells use dynamic changes in ensuring their progression and viability. There are also cancer hallmarks causing the initiation and progression of cancer. Following the alterations in the immune reactions and changes in blood glow, the cancer cells should be able to undergo continuous evolution within the malignant microenvironment (8, 9). This evolution by natural selection eventually results in adapted cancer cells that are resistant to drug and radiation therapies, increasing risk of death in advanced-stage of cancer (10). Therefore, recent studies have focused on understanding the mechanisms involved in cancer drug resistance (11), immune evasion (using novel strategies to improve immunotherapy) (12, 13) and radioresistance (14). Moreover, in addition to the transformation of normal cells into malignant cells, it has been demonstrated that lack of efficacy of immune cells in the elimination of newly generated tumor cells can also provide tumor progression (8, 15). Noteworthy, there is an increased risk of cancer in patients with compromised immune system and other factors including chronic stress, aging and chronic disease also play a key role.

One of the major hurdles in the treatment of cancer is chemoresistance that is similar to the resistance to drugs in infectious disease (16). Moreover, some early chemotherapy drugs including nitrogen mustard (17) and aminopterin (18) were successful, but the resistance and tumor recurrence impaired their efficacy, highlighting the need for more efficient antimicrobial therapies. medication number of factors including drug inactivation, increased drug efflux, and target modification, traditional small molecule therapies fail to produce the desired consequences and promising results (19, 20). Small molecule inhibitors (SMIs) become ineffective due to target protein alteration, including mutation and upregulation. Furthermore, the main aim of polychemotherapy was to eliminate tumors that had developed resistance to single-agent chemotherapy by combining drugs with different but complementary mechanisms of action. Such combination therapy has been beneficial in improving efficacy of treatments by blocking the regrowth of tumors at an early stage, which in turn led to the development of more potential and efficient regimens. However, it should be also noted that a combination of surgical resection, radiotherapy and polychemotherapy have not been able to completely eradicate tumors (16). Novel therapeutic approaches have been introduced to target certain features that can transform healthy cells and tissues into cancer. The use of nuclear receptors and tyrosine kinases as targets in cancer treatment has shown promising results. The initial successes of oestrogen receptor (ER) antagonists, BCR-ABL, HER2, and EGFR inhibitors brought about the development of agents targeting oncogenes and other cellular vulnerabilities (16). Monoclonal antibodies targeting PD-1/PD-L1 (21) and CTLA4 (22) have recently revolutionized cancer treatment, causing significant anti-tumor activity and, in some cases, complete treatment of cancer (23). However, cancer is considered as a complex biological disease and therefore, there is high risk of resistance to targeted and immunological treatments.

The cancer heterogeneity is considered as a main factor in cancer drug resistance that is a result of genetic alterations caused by mutational processes (24). These processes occur at different evolutionary speeds, from slow age-related mutations to frequent gene editing by APOBEC enzymes. There is an urgent need for the early therapeutic intervention, since large chromosomal abnormalities can be macro-evolutionary events causing resistance at a point of being irreversible (16). Another point worth noting is that larger tumors are associated with a higher risk of metastasis, and there is a nearly consistent link between tumor load and treatment (25). Although the early mathematical models of chemotherapy failed to highlight an inverse association, they did imply that a reduction in tumor burden may be achieved by combining various drugs, increasing the risk of disease eradication (26). Moreover, resistance is significantly affected by the tumor size, growth rate, and therapeutically-induced changes to growth kinetics. Tumors with low growth rates are typically incurable with cytotoxic chemotherapy or targeted therapies, while those growing at higher speeds can be sensitive to chemotherapy. There is a direct relationship between growth rate and tumour size (16). Therefore, chemotherapy has been mainly developed to target cancer cells with high growing rate. The rate of therapeutic resistance is frequently delayed, even as knowledge of cancer biology and the development of new drugs continues to advance.

## 2 Autophagy fundamentals and molecular machinery

### 2.1 Autophagy basics and molecular machinery

Autophagosome biogenesis is considered as a dynamic molecular process and it is a particular aspect of research (27). Moreover, autophagy demonstrates changes from intracellular space to the vacuole/lysosome lumen during the sequestration process, involving cytoplasm segregation. Phagophore is considered as a double-membrane compartment that releases the cargo into the lumen of degradative compartment, changing the topology during this process. A multitude of protein complexes and the mobilization of membrane reserves are involved in the short but dynamic process by which the phagophore develops into the autophagosome. After induction, nucleation, expansion, fusion, and cargo degradation/recycling, autophagosome biogenesis is complete. One important signaling route that varies as a cell’s extracellular environment changes is the target of rapamycin complex 1 (TORC1). The Atg1 kinase complex is activated when starvation begins an intracellular signaling cascade. The complex, consisting of Atg1, regulatory protein Atg13, and a scaffold subcomplex, is vital for the autophagy as it recruits other Atg proteins to the phagophore assembly site (PAS) and stimulates downstream targets through phosphorylation. It is worth noting that protein kinase A (PKA) is a negative regulator, while AMPK is a positive regulator (28–30). The autophagy machinery is initiated via nucleation that transfers a cluster of molecules to the phagophore, an active sequestering compartment. The proteins required for phagophore enlargement are recruited during this phase, which can be considered as an amplification event. Autophagy induction triggers the recruitment of the class III phosphotidylinositol 3-kinase (PtdIns3K) complex I to the PAS. The five proteins constituting the complex are Vps34, Vps15, Vps30/Atg6, Atg14, and Atg38 (31, 32). For the purpose of recruiting Atg8, Atg9, and Atg12 to the PAS, the proper localization of Atg proteins is required, and PtdIns3K is in charge of generating phosphatidylinositol-3-phosphate (PtdIns3P) (33, 34). The final stage of phagophore development is accompanied by the production of double-membraned vesicles known as autophagosomes during autophagy (35). Autophagosomes are essentially terminal compartments that fuse vacuoles, even if phagophores are transient. An autophagy dynamic consists of the phagophore’s production and attachment. The expansion of phagophores requires two ubiquitin-like (Ubl) conjugation systems, one requiring the Atg8 protein and the other the Atg12 protein (36). Although a number of proteins demonstrate structural similarities with ubiquitin, they are not homologs. The Atg12-Atg5-Atg16 complex is produced when Atg12 is conjugated to Atg5 by the action of the E1 and E2 enzymes Atg7 and Atg10, respectively. Due to its covalent bond with Atg8, the lipid phosphatidylethanolamine goes through a unique conjugation process. The development of Atg8-PE involves the protease Atg4, Atg7, and Atg3 as E1 and E2 enzymes (36, 37).

LC3 was initially identified in the rat brain as a light chain of microtubule-associated proteins 1A and 1B, including two isoforms, LC3A and LC3B. Initially, its function in cellular transport was poorly comprehended. Subsequently, Yoshimori’s team recognized LC3 as a pivotal element in autophagy machinery, improving the understanding of their functions. Recently, LC3C has been associated with autophagosome formation and COPII-mediated ER export via its interaction with TECPR2. Such finding demonstrates a possible link between the autophagy mechanism and the secretory route (38). Atg8s are a conserved eukaryotic protein family that evolved from a singular yeast gene into many subfamilies throughout mammals, plants, and protists. In mammals, Atg8 proteins are categorized into three subfamilies, LC3, GABARAP, and GATE-16 each possessing unique sequence signatures, gene copy variants, and evolutionary lineages. Humans have seven Atg8 genes distributed throughout the various subfamilies, but arthropods and insects demonstrate lineage-specific deletions and duplications. Atg8s possess an ubiquitin-like fold complemented by extra N-terminal α-helices, showing varying charges among subfamilies and can affect interactions. Notwithstanding sequence variability, their common structural characteristics support functions in autophagy via interactions with conjugation machinery, whilst divergent surfaces may provide specialized activities. This diversification presumably began with the emergence of multicellular organisms, and subsequent lineage-specific expansions, contractions, and isoform changes have affected the evolution and function of Atg8 across species (39).

The exact process by which these conjugation systems increase phagophore size is an active area of investigation and requires more understanding. Autophagosomes, sometimes known as being produced *de novo*, are different from vesicle budding, occurring during endocytosis and the secretory pathway. Secretory pathway vesicles demonstrate a consistent size and originate from pre-existing organelles. The cargo determines the size of the phagophore, which can be created by vesicular addition. As the phagophore grows, it is commonly believed that Atg9 acts as a membrane transporter (40). Atg9 is essential for phagophore expansion, highly mobile in the cytosol upon rapamycin treatment (41), and can binding with itself and transit to the PAS as part of a complex (42). Atg11, Atg23, and Atg27 are components of the Atg9 trafficking machinery. These components go from potential membrane donor locations to the PAS along with Atg9. Furthermore, the autophagosome attaches to the vacuole and releases its inner vesicle into the vacuole lumen to create an autophagic body during autophagy. The majority of complicated eukaryotic organisms do not possess autophagic bodies. Premature autophagosome fusion is prevented by regulatory systems, however the exact timing of fusion remains unknown (43), requiring further investigation. Deconjugation, a secondary cleavage event by Atg4, is required for autophagosome fusion and occurs in the context of Atg8-PE. Deconjugation may trigger disassembly of Atg proteins from the autophagosome, which precedes fusion. Other cellular processes, such as SNARE proteins and the homotypic fusion and vacuole protein sorting (HOPS) pathway, also use similar components for fusion (44). Moreover, once cargo reaches the vacuole, a putative lipase known as Atg15 breaks down the autophagic body membrane. Resident hydrolases then follow suit in breaking down the cargo (45, 46). Atg22 is one of several permeases that process macromolecule degradation and release their products back into the cytosol (47).

### 2.2 Autophagy in cancer drug resistance

Autophagy demonstrates a dual function in cancer growth and treatment resistance, that has been shown in various tumor types. In bladder cancer, the pan-Bcl-2 inhibitor (−)-gossypol induces both apoptosis and cytoprotective autophagy, with chemoresistant cells demonstrating increased basal autophagy that reduces apoptosis (48); genetic or pharmacological inhibition of autophagy sensitizes these resistant cells to Bcl-2 inhibition, highlighting the function of autophagy as a protective mechanism. In prostate cancer, STAT3 regulates chemotherapy-induced autophagy; its activation inhibits autophagy, aggravates mitochondrial damage, and diminishes cell viability, therefore associating STAT3 with mechanisms of chemoresistance (49). In hepatocellular carcinoma (HCC), mesenchymal stem cells (MSCs) subjected to inflammatory cytokines (IFN-γ and TNF-α) release TGF-β, enhancing protective autophagy in HCC cells, enhancing chemoresistance both *in vitro* and *in vivo*; inhibiting autophagy or silencing TGF-β negates this effect (50). Therefore, autophagy demonstrates association with oncogenic and inflammatory pathways and can function as a protective mechanism in causing tumor progression and chemoresistance. In addition, targeting autophagy along with oncogenic factors can improve efficacy of therapy.

Autophagy has become a pivotal mechanism contributing to the chemoresistance in several cancer types, including numerous molecular regulators and microenvironmental variables. In gastric cancer, elevated levels of autophagy-related gene 5 (ATG-5) and multidrug resistance protein MRP-1 are associated with advanced disease characteristics, diminished overall and disease-free survival, and chemotherapy resistance, highlighting their prognostic significance and functional role in protective autophagy (51). Transcription factor EB (TFEB), a principal regulator of lysosomal biogenesis, was demonstrated to promote doxorubicin (DOX) resistance in colon and cervical cancer cells by stimulating autophagy; its upregulation improved survival during chemotherapy, while TFEB knockdown or autophagy inhibition improved cytotoxicity of chemotherapy (52). In glioblastoma, autophagy facilitates chemoresistance by altering cellular metabolism, inducing quiescence, and enhancing survival, with transcriptome profiling uncovering both known oncogenic pathways and new genes possibly associated with glioblatoma development and therapeutic resistance (53). In addition to intrinsic tumor cell mechanisms, the tumor microenvironment (TME) affects autophagy-related chemoresistance, as demonstrated in colorectal cancer, where the prevalence of Fusobacterium nucleatum correlated with recurrence and treatment failure. Mechanistically, this gut microbe stimulated autophagy via TLR4/MYD88 signaling and microRNA modulation, consequently diminishing the effectiveness of chemotherapy (54). Therefore, autophagy has a vital and complicated survival function in cancer and this can be modulated by tumor genetic factors, transcriptional regulators, metabolic adaptations and microbial interactions, providing the fact that its suppression can improve efficacy of chemotherapy.

Recent studies highlight autophagy as a central mechanism driving chemoresistance across diverse cancers, with distinct molecular mediators linking survival pathways to therapy failure. Cisplatin has been demonstrated to upregulate GFRA1 in osteosarcoma that can accelerate AMPK-mediated autophagy through SRC phosphorylation to reduce apoptosis and increase cancer growth. Moreover, autophagy suppression can improve cisplatin sensitivity (55). The mesenchymal stromal cells have been shown to stimulate ATG7-related autophagy in leukemia to support against cytarabine and idarubicin, while whereas ATG7 silencing in both AML cells and stroma disrupts BCL2 family signaling, upregulates pro-apoptotic NOXA, and improves survival in mouse models, highlighting autophagy inhibition as a therapeutic avenue (56). Colon cancer stem cells also utilize autophagy for resistance: CD^44+^CD^24+^Cdx^1+^ cells demonstrate high Bcl-2 and LC3-II/I ratios, resisting paclitaxel-induced death, while Cdx1 silencing or lysosomal inhibition sensitizes them, contrasting with p53-driven apoptosis and autophagy suppression in CD^44+^CD^24+^p53wt cells (57). In addition, epithelial carcinoma cells exposed to cisplatin, 5-flourouracil and docetaxel demonstrate a drug resistance phenotype that has been shown by oxidative damage adaptation through Nrf2 upregulation and autophagy-mediated clearance of toxic p62 aggregates (58); disrupting this pathway by inhibiting autophagy or altering p62 function recovers chemosensitivity. Hence, it can be concluded that how cancer cells leverage autophagy through GFRA1 signaling, ATG7 regulation, Cdx1-driven stemness, or p62 homeostasis to evade chemotherapy, providing autophagy and its molecular regulators as promising therapeutic targets to overcome resistance.

Epithelial ovarian cancer (EOC) is one of the most aggressive gynecological cancers, demonstrating significant mortality mostly due to tumor recurrence after treatment. A small subgroup of cancer stem cells (CSC) is believed to facilitate development and recurrence, demonstrating resilience to starvation and chemotherapy. Drug resistance has been strongly associated with the induction of autophagy. *In vitro* and *in vivo* research demonstrated that ovarian CSCs, characterized by CD44/CD117 co-expression, have elevated basal autophagy compared to the non-stem counterparts. Inhibition of autophagy, either chloroquine administration or CRISPR/Cas9-induced ATG5 deletion, diminished CSC viability, spheroid forming ability, and tumorigenic potential. Furthermore, the combination of autophagy suppression and carboplatin therapy improved synergistic effects, reducing CSC activities and tumor proliferation. These evidences highlight the vital function of autophagy in the maintenance of CSCs and demonstrate that concurrently targeting this system with chemotherapy may be a viable approach to surmount resistance and revert recurrence (59).

**Figure 1:**
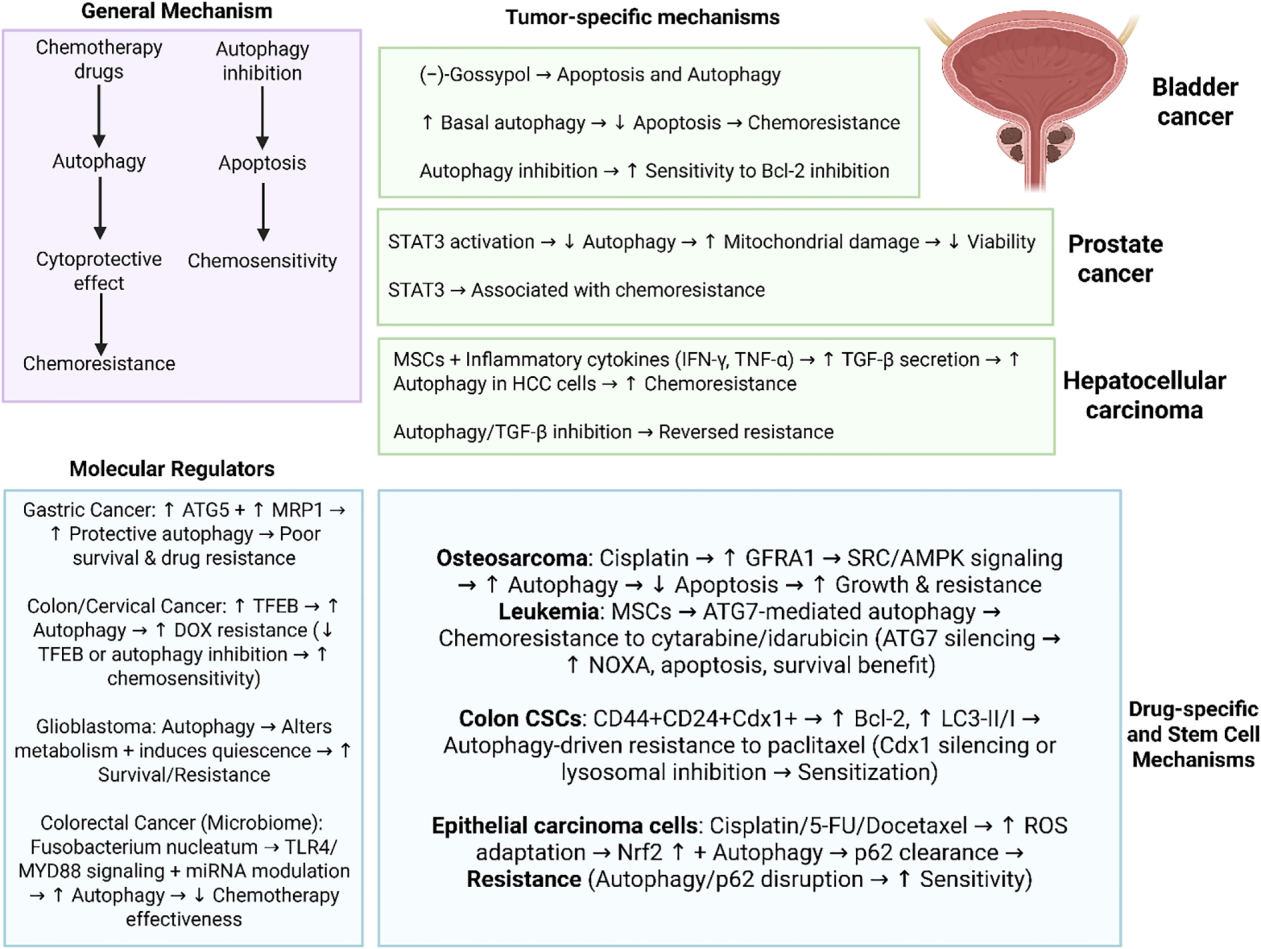
Autophagy in cancer drug resistance. Overall, there are two concepts of autophagy in chemotherapy including protective autophagy and cytotoxic autophagy. Notably, there are several important molecular regulators of autophagy including ATG5, TFEB, miRNAs and TLR4/MYD88 axis that their abnormal expression can affect autophagy in the regulation of chemotherapy response. Moreover, autophagy appears to be context-dependent and cancer specific such as role of autophagy in bladder cancer, STAT3-driven autophagy regulation in prostate tumor and interaction with TGF in hepatocellular carcinoma. Therefore, autophagy demonstrates interactions with specific pathways and molecular factors in each specific type of cancer that can be considered as promising therapeutic targets. (Biorender.com).

One of the main features of solid tumors is hypoxia, enhancing the activation of adaptive mechanisms including autophagy to enhance survival. In HCC, autophagy can be considered as a protective mechanism accelerating therapeutic resistance in hypoxic TME. In comparison to the normal conditions, hypoxia has been demonstrated to decrease chemotherapy-mediated cell death through apoptosis evasion. The autophagy suppression with 3-MA or Beclin-1 siRNA reversed this event, decreasing cancer drug resistance. Autophagy has been able to decrease response of hepatoma cells to the anti-cancer drugs through reducing apoptosis (60). **Figure 1** highlights the role of autophagy in cancer drug resistance and related pathways.

## 3 Doxorubicin mechanisms of action and resistance

The damage to cell membrane DNA and various cellular proteins caused by free radical generation and intercalation into cellular DNA, consequently disrupting DNA repair specifically mediated by topoisomerase IIα (TOP2A), are the two most prominent and widely accepted mechanisms linked to DOX action (61, 62). Moreover, reactive oxygen species (ROS) are produced during the conversion of DOX to the unstable intermediate metabolite known as semiquinone. The semiquinone is then transformed back to DOX. The peroxidation of lipids causes the cell membrane damage, DNA damage, and finally the start of apoptosis due to the generation of free radicals (63). A group of genes known as free radical generators (NADH dehydrogenase, NO synthase, and xanthine oxidase) and a group of genes titled free radical deactivators (antioxidants, specifically glutathione peroxidase, superoxide dismutase, and catalase) constitute the corresponding genetic repertoire (64, 65). The second method suggests that DOX enters the target cell’s nucleus, intercalates with the host DNA, and then targets TOP2A (66). DNA strand breaks (DSBs) are generated and repaired by TOP2A, which is also in charge of releasing entangled DNA (67). By blocking TOP2A’s action, DOX blocks the repair process and causes a high number of double-strand breaks to form. Damaged DNA breaks trigger the caspase-dependent apoptosis cascade by activating p53 and FOXO3. The impact on the cell death demonstrated by various Bcl2 protein members is distinct (68). DOX may also stimulate apoptosis by blocking DNA and RNA synthesis and enhancing mitochondrial ROS generation (62). DOX also controls to activate p53, a tumor suppressor that tries to shield cells from specific changes that can cause tumors (69). DOX has demonstrated promising consequences in suppressing the advancement of numerous malignancies, such as gynecologic, brain, and lung cancers (70–73). The high anti-cancer activity of DOX has resulted in its frequent application as chemotherapy regimen. On the other hand, cancer cells can establish resistance to chemotherapy with repeated administration (74). Therefore, it is suggested to use short and fractioned administration. The development of DOX resistance is affected by both intrinsic and extrinsic factors, as well as the TME (75–77). In addition, recent developments in genetics and bioinformatics have uncovered multiple molecular pathways inducing DOX resistance. These pathways include up-regulation of P-glycoprotein, activation of tumor-promoter factors, inhibition of apoptosis, and stimulation of protective autophagy (78–81). Therefore, more focus should be directed towards the critical factors involved in DOX resistance.

There have been new approaches in overcoming DOX resistance in human cancers. In order to maximize the efficacy of DOX chemotherapy and improve sensitivity to apoptosis, genetic tools such as siRNA or shRNA are utilized in combination cancer therapy (82–84). Nanocarriers can also be used for combined application of DOX with genetic tools or anti-cancer compounds (85). Nanostructures for co-delivery enhance intracellular accumulation, provide endosomal escape, and protect nucleic acids from degradation by RNase enzymes. In addition, nanostructures prolong blood circulation time (86–90). Therefore, one of the promising strategies is the application of nanoparticles for the targeted delivery of chemotherapy drugs and combination with other anti-cancer drugs or genetic tools. Moreover, a small amount of DOX is loaded on nanoparticles for cancer therapy that enhances the potential in tumor suppression and reducing the risk of cancer drug resistance.

In addition, it has been shown that non-coding RNAs participate in the development of DOX resistance in human cancers (91). Several factors participate in cancer drug resistance such as p53 mutation (92). Among the many factors that contribute to the development of cancer, DOX blocks the cell cycle (91). DOX causes p53 mutations and MDR after long-term exposure. It is also possible for chemotherapeutic drugs to develop cross-resistance (multidrug resistance) (93). DOX resistance following p53 mutations is attributed to the up-regulation of P-glycoprotein (P-gp), a drug efflux transporter involved in pumping out DOX from cancer cells (93). Drug transporters and DOX resistance can be affected by several molecular pathways; for example, Nrf2 can inhibit DOX accumulation in cells by increasing ABCB1 expression (94). Therefore, the future studies can also focus on targeting epigenetic factors, especially non-coding RNAs in overcoming DOX resistance. Moreover, inhibition of drug efflux transporter activity on the surface of cancer cells can further suppress DOX resistance.

Other factors for overcoming DOX depends on targeting special organelles in the cells such as mitochondria due to its function in apoptosis regulation and also, impact of ROS on this organelle (95, 96). The dysfunction of mitochondria can cause apoptosis and decease the balance of energy for the growth of tumor cells that can be mediated through the downregulation of mitochondrial transcription factor A. Therefore, more focus has been directed towards the function of mitochondria in cancer (97, 98). Reduced expression of microRNA-125b causes apoptotic cell death in DOX-resistant breast cancer cells (99). Under hypoxic conditions, sirtuin 1 and AMP-activated protein kinase activity are inhibited, leading to DOX resistance (100). Exposure to DOX affects the expression of structural and functional mitochondrial genes, affecting the overall response to DOX (101). As a result, targeting organelles should be considered as a promising strategy in reversing DOX resistance and due to the versatile function of mitochondria in cell death, a major focus should be directed on this organelle.

The clinical studies have also evaluated the function and anti-cancer activity of DOX. Notably, DOX resistance has been a challenging issue in clinical studies. The combination of DOX with VX-710 can improve therapy response, stabilize disease, and promote overall cancer patient survival (102). In order to improve the function of DOX in cancer therapy, it is suggested to use polychemotherapy (103, 104). A liposomal form of DOX with valspodar has been used to improve its efficacy in cancer therapy, but does not affect its toxicity against cancer cells (105). Another point to consider is that the involvement of non-coding RNAs in cancer cell metastasis suppression and DOX sensitivity is vital in DOX resistance. The non-coding RNAs are able to regulate various biological mechanisms in tumor cells (106, 107). DOX resistance has been mediated via the down-regulation of miRNA-125b by SMYD2 in renal cancer cells (108). Therefore, these discussions highlight the fact that various biological mechanisms and molecular pathways participate in the development of DOX resistance. Therefore, the present review has been aimed to evaluate the function of autophagy in the regulation of DOX resistance and understanding its interaction with other biological mechanisms and molecular pathways that will be discussed specifically in the upcoming sections.

## 4 Autophagy-doxorubicin molecular interactions

The hsa-circ-0092276 has been recognized as significantly upregulated in DOX-resistant breast cancer cells, demonstrating its involvement in chemotherapy resistance. DOX-resistant breast cancer cell lines (MCF-7/DOX and MDA-MB-468/DOX) were developed, showing significantly elevated half-maximal inhibitory concentration (IC_50_) values compared to their parental lines, MCF-7 and MDA-MB-468. The resistant cells demonstrated increased levels of the drug resistance-related protein MDR1. The expression of hsa_circ_0092276 was significantly elevated in MCF-7/DOX and MDA-MB-468/DOX cells relative to parental cells. The overexpression of hsa_circ_0092276 enhanced proliferation, elevated LC3-II/LC3-I and Beclin-1 expression, and decreased apoptosis in breast cancer cells. Such events were reversed by 3-methyladenine, an inhibitor of autophagy. Mechanistically, hsa_circ_0092276 modulated the ATG7 through the sequestration of miR-384. The up-regulation of hsa_circ_0092276 enhanced autophagy and proliferation while inhibiting apoptosis, results that were counteracted by the overexpression of miR-384 or the silencing of ATG7. Moreover, the transplanting of MCF-7 cells transfected with LV-circ_0092276 into mice enhanced autophagy and tumor proliferation (109). This study provides a promising fact in which the interaction between non-coding RNAs including circRNAs and miRNAs can affect the ATGs to modulate autophagy in the regulation of DOX resistance. Regarding this, another study was also focused on the role of lncRNAs in the regulation DOX resistance. A multitude of natural antisense lncRNAs have demonstrated significant roles in cancer biology. FOXC2-AS1 and its antisense transcript FOXC2 are significantly increased in DOX-resistant osteosarcoma cell lines and tissues, correlate with worse prognosis, and facilitate DOX resistance. The two transcripts are mainly located in the cytoplasm and develop an RNA–RNA double-stranded structure in the overlapping region, which is vital for FOXC2-AS1 to modulate FOXC2 expression at both transcriptional and post-transcriptional stages. Moreover, the transcription factor FOXC2 enhances DOX resistance by upregulating the expression of the multi-drug resistance gene ABCB1, a process that FOXC2-AS1 also uses. FOXC2-AS1 together enhances DOX resistance in osteosarcoma through the upregulation of FOXC2, which in turn boosts ABCB1 expression (110). The other aspects into the role of non-coding RNAs in DOX resistance could evaluate additional regulatory factors beyond circRNAs and natural antisense lncRNAs. The systematic studies on other classes of lncRNAs, circRNAs, and miRNAs could help uncover novel RNA–RNA or RNA–protein interaction networks that modulate autophagy, apoptosis, and drug efflux pathways. Furthermore, examining whether competing endogenous RNA (ceRNA) networks involving multiple non-coding RNAs synergistically regulate ATGs, ABC transporters, or apoptosis regulators could provide better insights. It would also be valuable to investigate tissue-specific or tumor subtype-specific expression patterns of ncRNAs, as well as their interactions with epigenetic modifiers, transcription factors, and signaling pathways such as PI3K/AKT or MAPK in the context of chemoresistance. In addition, *in vivo* studies and patient-derived xenografts could validate the clinical significance of these ncRNA-mediated mechanisms, potentially identifying biomarkers for predicting DOX response and new therapeutic targets to overcome resistance.

One of the promising compounds for the treatment of HCC is DOX, but its long-term efficacy has been comprised by the emergence of acquired resistance. Autophagy, a conserved catabolic process for the cellular preservation and environmental adaptability, has been identified as a possible therapeutic target to address DOX resistance. The function of miR-223 in modulating DOX-induced autophagy and drug sensitivity was examined in four transfected human HCC cell lines, with *in vivo* validation conducted using a mouse xenograft model of HCC. miR-223 was observed to be expressed at reduced levels in DOX-treated HCC cells, whereas the upregulation of miR-223 impeded DOX-induced autophagy, resulting in the chemoresistance. The inhibition of autophagic flow by chloroquine negated the efficacy of a miR-223 inhibitor in reducing DOX sensitivity in HCC cells. FOXO3a was recognized as a direct downstream target of miR-223 and functioned as the principal mediator of its regulatory impact on DOX-induced autophagy and chemoresistance. In xenograft models of HCC, agomiR-223 administration increased sensitivity to DOX (111). In order to expand the idea to the other solid tumors, it is also suggested to evaluate the miR-223/FOXO3a axis in other cancers and broaden the idea. After these investigations, it can be considered that this axis is of importance in solid tumors for causing DOX resistance. Therefore, it should be targeted for reversing chemoresistance in cancer patients.

There are studies emphasize the impact of various autophagy mechanisms and microenvironmental factors on DOX resistance in cancer cells. In colorectal cancer stem cells (CSCs), DOX resistance was specifically associated with mitophagy rather than general autophagy (112). CSCs demonstrated reduced mitochondrial superoxide levels, elevated BNIP3L expression, and enhanced mitophagy compared to the parental cells, while BNIP3L silencing rendered them more susceptible to DOX, emphasizing the protective function of mitophagy. Conversely, in breast cancer cells, the tumor microenvironment significantly influenced the response to DOX. Under 3D laminin-rich ECM conditions, MCF-7 cells demonstrated increased sensitivity to DOX but demonstrated diminished activation of p53-DRAM-1-mediated autophagy, demonstrating that the lack of this autophagy pathway enhanced cytotoxicity (113). In 2D cultures, cells maintained the p53-DRAM-1 axis, with autophagy serving as a cytoprotective mechanism; reduction of p53 or DRAM-1 increased DOX-induced cytotoxicity in 2D cultures, not in 3D cultures. Moreover, combinatorial targeting of the ubiquitin–proteasome system (UPS) and autophagy in breast cancer highlighted an additional perspective mechanism. The concurrent administration of DOX and ixazomib stimulated substantial autophagy, yet its inhibition via hydroxychloroquine significantly enhanced cytotoxicity (114). Furthermore, the triple treatment synergistically impaired growth in both MCF-7 and MDA-MB-231 cells while resulting in the accumulation of ubiquitinated proteins. Hence, these findings demonstrate that resistance to DOX develops via distinct, context-dependent survival mechanisms mitophagy in CSCs, p53-DRAM-1–mediated autophagy in monolayer breast cancer cells, and UPS–autophagy interactions in breast cancer more generally, emphasizing the necessity of targeting specific adaptive pathways based on cell type and microenvironment.

Since previous studies highlight the function of mitophagy in the modulation of DOX resistance, it would be of importance to understand some of the mechanisms related to the mitophagy in tumorigenesis. Breast cancer and HCC demonstrate resistance to chemotherapy, with mitophagy, a selective autophagic mechanism that targets damaged mitochondria, playing a vital role in this resistance. In breast cancer, specifically in luminal A MCF7 cells and triple-negative MDA-MB-231 cells, DOX was demonstrated to activate the canonical PINK1/Parkin-mediated mitophagy pathway (115). Inhibition of this pathway via miRNA-218-5p, which targets Parkin, enhanced sensitivity to DOX, highlighting the protective function of mitophagy against chemotherapy. In HCC, the efficacy of doxorubicin-induced immunogenic cell death (ICD) was limited; however, its effectiveness was augmented by the addition of icaritin, which stimulated both mitophagy and apoptosis to enhance ICD (116). When co-administered with DOX via targeted PLGA-PEG-AEAA nanoparticles, the treatment restructured the immunosuppressive TME, stimulated lasting immune memory, and improved survival, particularly in conjunction with lenvatinib. Cisplatin treatment in HCC initiated DRP1-dependent mitophagy. Pharmacological inhibition of DRP1 using Mdivi-1 obstructed mitophagy, increased apoptosis via Bax upregulation, Bcl-xL downregulation, and cytochrome c release, and acted synergistically with cisplatin to inhibit tumor growth *in vivo* (117). These findings highlight mitophagy as a dual-faceted mechanism: it promotes tumor survival and chemoresistance, yet its modulation through inhibition to enhance chemotherapy sensitivity or strategic induction to increase immunogenic cell death, presents promising therapeutic strategies for both breast cancer and hepatocellular carcinoma. Beyond the canonical PINK1/Parkin and DRP1-dependent pathways, other aspects of mitophagy in DOX resistance include the involvement of receptor-mediated mitophagy regulators such as BNIP3, NIX, and FUNDC1, the intricate crosstalk between mitophagy and apoptosis in controlling mitochondrial clearance versus pro-apoptotic signaling, and the impact of the TME, particularly hypoxia and nutrient stress, in shaping mitophagy-mediated chemoresistance. Additionally, regulation by a broader spectrum of non-coding RNAs (miRNAs, lncRNAs, and circRNAs) and the role of mitophagy in metabolic reprogramming to support tumor survival further underscore its complexity. Therapeutically, strategies combining mitophagy modulation with immune checkpoint inhibitors, metabolic modulators, or nanoparticle-based drug delivery may provide new avenues to overcome DOX resistance in breast cancer and HCC.

The therapeutic use of anthracyclines in cancer treatment is limited by dose-dependent cardiotoxicity, characterized by damage and death of cardiomyocytes. Anthracyclines, including DOX, have demonstrated an impact on protein degradation pathways in adult cardiomyocytes. In long-term cultured adult rat cardiomyocytes, DOX administration led to the accumulation of poly-ubiquitinated proteins, an elevation in cathepsin-D-positive lysosomes, and destruction of myofibrils. The chymotrypsin-like activity of the proteasome first enhanced but was later suppressed during a 48-hour duration. Increased dosages of DOX resulted in the downregulation of 20S proteasome proteins. The expression of MURF-1, a ubiquitin ligase that selectively targets myofibrillar proteins, was suppressed at all doses examined. DOX treatment also stimulated LC3-positive puncta and increased levels of LC3-I and LC3-II proteins in a dose-dependent manner, as shown by lentiviral production of green fluorescent protein conjugated to LC3 and live imaging. The administration of the lysosomotropic agent chloroquine resulted in the accumulation of autophagosomes, a phenomenon further enhanced by simultaneous exposure to DOX, signifying an elevation in autophagic flux. DOX suppressed the protein degradation mechanisms in cardiomyocytes, resulting in the accumulation of poly-ubiquitinated proteins and autophagosomes. While autophagy was originally activated as a compensatory response to the cytotoxic stress, extended exposure and elevated dosages led to apoptosis and necrosis. This mechanism may aggravate the delayed cardiotoxic effects of anthracyclines by changing the senescence of postmitotic cardiomyocytes and increasing the susceptibility of the aging heart during anthracycline-based cancer treatment (118). This study provides promising results about the fact that it is not possible to increase DOX dosage, as it causes toxicity, especially on heart. Therefore, new strategies should be developed in reversing DOX resistance and maintaining DOX dosage to the optimal levels.

As it was mentioned, the application of DOX for cancer therapy has faced a number of challenges that in addition to toxicity, the most important one is resistance and autophagy has been considered as a promising biological mechanisms in cancer drug resistance. In triple-negative breast cancer (MDA-MB-231 cells), the suppression of autophagy via 3-methyladenine sensitized the cells to DOX, resulting in synergistic cytotoxic effects, downregulation of ATGs, and a transition in cell death modality from apoptosis to necroptosis, demonstrate that autophagy blockade enhances DOX efficacy (119). In MCF-7 breast cancer cells, the development of resistance to DOX was linked to the redistribution of the drug to the perinuclear region, its colocalization with lysosomes and autophagosomes, and elevated levels of autophagy markers such as LC3-II and p62 (120). Furthermore, the inhibition of autophagy using chloroquine restored drug sensitivity, suggesting a survival mechanism differing from traditional starvation-induced autophagy. Resistin, an adipokine associated with obesity, exacerbates resistance by activating the AMPK/mTOR/ULK1 and JNK signaling pathways, thereby enhancing autophagy flux and reducing DOX-induced apoptosis (121). Inhibition of autophagy abrogates its pro-survival effect, indicating that resistin antagonism may serve as a viable therapeutic strategy. In osteosarcoma (U2OS and Saos-2 cells), DOX triggered both apoptosis and autophagy, the latter serving as a protective mechanism; suppression of autophagy with Atg7 siRNA or 3-methyladenine significantly enhanced apoptosis and accelerated DOX’s anticancer efficacy (122). Across several cancer models, these findings highlight autophagy as a critical modulator of DOX resistance and propose that the combination of DOX with autophagy inhibitors may yield a more efficacious treatment approach to surmount chemoresistance.

Pancreatic cancer ranks as the fourth foremost cause of cancer-related mortality globally, and existing chemotherapeutic treatments offer minimal advantage due to drug resistance, highlighting the pressing want for new efficacious techniques. Deguelin, a natural chemopreventive agent, demonstrates significant antiproliferative effects in solid tumors by inducing cell death, however its exact molecular pathways are not fully elucidated. Deguelin has been demonstrated to inhibit autophagy and induce apoptosis in pancreatic cancer cells. Given that DOX-induced autophagy functions as a protective mechanism in pancreatic cancer cells, the inhibition of autophagy by chloroquine or the silencing of autophagy protein 5 significantly increased DOX-induced cell mortality. Similarly, deguelin-induced suppression of autophagy enhanced the sensitivity of pancreatic cancer cell types to DOX. The findings demonstrate that deguelin possesses significant anticancer efficacy against pancreatic cancer and amplifies the therapeutic benefits of DOX (123). In addition to the role of autophagy in mediating DOX resistance, other aspects that can be considered include the interplay between the TME and drug response, as factors such as hypoxia, stromal interactions, and immune modulation may affect both autophagy and chemoresistance. The contribution of CSCs, which are often more resistant to DOX, should also be evaluated, as autophagy has been implicated in their survival and self-renewal. Furthermore, genetic and epigenetic alterations, including non-coding RNAs (such as miRNAs and lncRNAs), could regulate autophagy pathways and affect DOX sensitivity. Exploring metabolic reprogramming, including changes in glycolysis and mitochondrial dynamics, may provide additional insight since autophagy intersects with these processes. In addition, the potential for combination therapies that integrate autophagy inhibitors with targeted therapies, immunotherapies, or nanoparticles for more effective DOX delivery represents an important avenue to overcome resistance and enhance therapeutic efficacy.

Chemoresistance continues to be a challenging issue and the function of DOX-driven autophagy has been of importance. In HCC, DOX administration induces HMGB1 expression and its cytoplasmic translocation, thereby activating autophagy through the AMPK/mTOR pathway, protecting cells from apoptosis and increases resistance; suppression of HMGB1 or autophagy renders both parental and resistant HCC cells more susceptible to DOX (124). In gastrointestinal cancers, DOX induces autophagy via ROS generation, alongside Beclin1 upregulation, Bcl2 downregulation, AMPK and JNK activation, and Akt inhibition, whereas antioxidant pretreatment mitigates these effects; resistant cells demonstrate reduced ROS-dependent apoptosis, associated with elevated expression of AKR1B10 and AKR1C3, whose inhibition reinstates DOX sensitivity (125). MAGEA6 is significantly expressed in resistant tumors of triple-negative breast cancer (TNBC) and affects doxorubicin DOX resistance through the modulation of autophagy and ferroptosis (126). Silencing MAGEA6 diminishes AMPKα1 ubiquitination, activates AMPK signaling, promotes autophagy, and induces ferroptosis, consequently enhancing DOX sensitivity. In breast cancer MCF7 cells, the development of a resistant subline (MCF7.res) demonstrated a 7.1-fold resistance to DOX, characterized by increased LC3-II expression and lysosomal mass, signifying increased autophagic flux. Inhibition of autophagy using chloroquine reversed this resistance and recovered apoptosis (127). These studies emphasize the vital function of autophagy, frequently facilitated by pathways including HMGB1/AMPK/mTOR, ROS signaling, and MAGEA6/AMPK/SLC7A11, in the emergence of DOX resistance in various cancers, and highlight the therapeutic promise of targeting autophagy and its associated regulators to surmount chemoresistance.

Drug resistance is a significant challenge in cancer chemotherapy, since cancer cells frequently utilize autophagy to endure therapeutic stress, thereby reducing treatment effectiveness. Therefore, it can be concluded that autophagy can function as a pro-survival mechanisms in chemoresistance.Biomarkers that particularly signify autophagy-mediated medication resistance are inadequately characterized. Lipid rafts, or cholesterol-enriched membrane micro-domains (CEMMs), have been recognized for their new function in autophagosome formation and DOX resistance in breast cancers. CEMMs are vital for the interaction between VAMP3 and the cholesterol-binding SNARE protein syntaxin 6 (STX6). Disruption of CEMMs results in the release of VAMP3 from STX6, therefore enabling the transport of ATG16L1-containing vesicles to recycling endosomes and enhancing autophagosome biogenesis. Decreased expression of the CEMM marker CAV1 has been highlighted in breast cancer patients, and CEMM deficiency-induced autophagy is associated with DOX resistance, which can be mitigated by autophagy suppression. This model demonstrates that CEMMs in recycling endosomes maintain the VAMP3–STX6 relationship and serve as barriers to restrict VAMP3 activity in autophagic vesicle fusion, whereas CEMM loss promotes autophagosome production and contributes to DOX resistance in breast cancers (128).

Breast cancer is the most prevalent neoplasm among women. Chemotherapy is the principal systemic treatment; yet, its efficacy is prevented by chemoresistance. Autophagy has been demonstrated to increase tumor cell survival during therapeutic stress, therefore enhancing chemoresistance. The function of lysosomes in the completion of autophagy has not been fully elucidated regarding its contribution to autophagy-related chemoresistance. The lysosomal gene ATP6AP1 has been identified as a possible regulator of this mechanism, perhaps increasing chemoresistance in breast cancer through the upregulation of autophagic flux. The toxic effects of DOX on cell viability were assessed by cytotoxicity tests, flow cytometry, and lactate dehydrogenase (LDH) release in several breast cancer cell lines. Autophagic flow was assessed using western blotting and mRFP-GFP-LC3 fluorescence microscopy. Breast cancer cells were transduced with shRNA lentivirus aimed at ATP6AP1 to evaluate its function in DOX-induced cytotoxicity. The expression levels of ATP6AP1 and their correlation with prognosis were examined utilizing public databases and immunohistochemistry. The cell death produced by DOX in breast cancer cells was negatively correlated with increased autophagic flux and lysosomal acidification. ATP6AP1, a lysosomal gene implicated in autophagic mechanisms, was observed to be increased in breast cancer tissues. Inhibition of ATP6AP1 diminished autophagy-related DOX resistance by decreasing autophagic flux and lysosomal acidification. Data from public databases and clinical cohorts indicated that elevated ATP6AP1 expression was associated with a reduced response to DOX-based neoadjuvant chemotherapy (NAC) and a worse prognosis. The cytotoxicity of DOX in breast cancer is affected by autophagic flux. ATP6AP1 enhances autolysosome acidification, hence providing DOX resistance and leading to unfavorable treatment results (129). The clinical importance of these findings is in the recognition that DOX-induced autophagy functions as a critical pro-survival mechanism that emphasizes the therapeutic efficacy of chemotherapy across multiple cancers. By activating pathways such as HMGB1/AMPK/mTOR in HCC, ROS–Beclin1 signaling in gastrointestinal cancers, MAGEA6/AMPK-driven autophagy and ferroptosis regulation in TNBC, and ATP6AP1-mediated lysosomal acidification in breast cancer, tumor cells can escape DOX-induced apoptosis and develop resistance. Additionally, lipid raft–mediated regulation of autophagosome formation in breast cancer further highlights the complexity of this adaptive response. Clinically, these mechanisms emphasize the potential value of combining DOX with autophagy inhibitors, lysosomal function modulators, or regulators of lipid raft integrity to overcome resistance. Moreover, biomarkers such as HMGB1, MAGEA6, ATP6AP1, and CEMM markers such as CAV1 may serve as predictors of treatment response and prognosis, enabling more personalized therapeutic strategies. Thus, targeting autophagy-related pathways provides significant translational promise for improving DOX-based chemotherapy outcomes in resistant malignancies.

In spite of the development of various chemotherapy regimens in the treatment of breast cancer, the drug resistance has been a problematic issue in the treatment of this malignant disease. Enhancing tumor cell susceptibility to chemotherapeutic drugs is essential for enhancing treatment results. In MDA-MB-231 human breast cancer cells treated with DOX, an increase in HO-1 expression was observed, and these cells demonstrated decreased sensitivity to DOX. Inhibition of HO-1 expression significantly enhanced DOX-induced cytotoxicity in MDA-MB-231 cells, demonstrating that HO-1 is a vital mediator of drug resistance. DOX treatment was observed to induce cytoprotective autophagic flux in MDA-MB-231 cells, contingent upon HO-1 expression. The increase of HO-1 necessitated the activation of the Src/STAT3 signaling pathway. Inhibition of Src or STAT3 decreased HO-1 expression and autophagy, thereby increasing the chemosensitivity of MDA-MB-231 cells. Subsequent investigation with the MDA-MB-468 breast cancer cell line, which has a comparable phenotype to MDA-MB-231, validated that the activation of the Src/STAT3/HO-1/autophagy pathway plays a role in DOX resistance. The data suggest that the Src/STAT3-mediated activation of HO-1 safeguards certain breast cancer subtypes from DOX-induced cytotoxicity by enhancing autophagy. Targeting this signaling system may serve as a viable therapeutic option to mitigate DOX resistance in breast cancer (130).

Understanding the autophagy mechanisms and related molecular pathways appears to be vital for highlighting the DOX resistance in osteosarcoma. In osteosarcoma, the overexpression of CXCR4 provides resistance by maintaining P-glycoprotein and PI3K/AKT/mTOR pathways, whereas the silencing of CXCR4 amplifies DOX-induced apoptosis via autophagic cell death (131); significantly, the inhibition of autophagy with bafilomycin abrogated this sensitization, and the CXCR4 antagonist AMD3100 demonstrated a synergistic effect with DOX *in vivo*. Amino acid deprivation in breast cancer enhanced autophagy and diminished apoptosis in normal MCF12A cells, thereby protecting them from DOX toxicity; conversely, metastatic MDA-MB-231 cells demonstrated no such advantage (132). Additionally, short-term starvation *in vivo* extended survival in treated mice, demonstrating the context-dependent protective effects of autophagy induction. In leukemia (K562) cells deficient in p53 and p16, DO-induced senescence correlated with the upregulation of miR-375, the repression of 14-3-3zeta and SP1, and the enhanced expression of autophagy genes (ATG9B, ATG18), highlighting a p53/p16-independent senescence mechanism linked to the initiation of autophagy (133). In cervical and liver cancer models demonstrating acquired DOX resistance, resistant cells adapted by diminishing energy metabolism and chromatin acetylation, activating pro-survival autophagy, and decelerating proliferation; the inhibition of autophagy or pre-treatment with histone deacetylase inhibitors (HDACi) resensitized these cells to DOX (134). These studies highlight autophagy’s dual function in chemotherapy, occasionally facilitating cancer cell death and at other times supporting survival and demonstrate that customized approaches targeting CXCR4, autophagy flux, or epigenetic regulators may aid in overcoming resistance while safeguarding healthy tissues.

Autophagy, a critical mechanism in cancer biology, is intricately associated with tumor growth and the emergence of treatment resistance. Traditional methods for assessing autophagy frequently demonstrate invasiveness and temporal constraints, diminishing their effectiveness in preclinical drug assessment. To tackle these issues, a non-invasive autophagy detection system (NIADS-autophagy), also known as the G-cleave LC3B biosensor, was developed by integrating a split-luciferase biosensor with an LC3B cleavage sequence. This method immediately identified classical autophagy inducers, including Earle’s Balanced Salt Solution and serum deprivation, via protease-mediated degradation pathways. The specificity of the G-cleave LC3B biosensor was confirmed using CRISPR-mediated deletion of the essential autophagy regulator ATG4B, leading to diminished luciferase activity in MDA-MB-231 breast cancer cells. Robust concordance was demonstrated between the biosensor and traditional autophagy markers, encompassing LC3B lipidation, SQSTM1 degradation, and puncta formation experiments. The G-cleave LC3B biosensor demonstrated that resveratrol acts as a synergistic enhancer, significantly augmenting apoptosis in MDA-MB-231 cells when administered with doxorubicin therapy. The luminescence-based G-cleave LC3B biosensor provides a quick and dependable method for evaluating autophagy activity, facilitating high-throughput analysis of autophagy-related anticancer approaches across various tumor types (135).

Breast cancer continues to be the predominant malignancy in women, with over 220,000 new cases and 41,000 fatalities per year in the United States. The emergence of resistance to chemotherapeutic drugs significantly contributes to recurrence and death, highlighting the significant necessity to enhance understanding of disease biology and resistance mechanisms to refine current therapies and formulate novel treatment options. Autophagy has achieved significant interest because to its activation by several anticancer modalities, including chemotherapy, antiestrogen therapies such as tamoxifen, and radiation treatment. This highly regulated, lysosome-dependent mechanism degrades misfolded proteins, macromolecules, and organelles in response to stressors such as nutrient deficiency, oxidative stress, and hypoxia, potentially resulting in either cell survival or caspase-independent autophagic cell death, contingent upon its intensity and duration. DOX, a prevalent chemotherapeutic agent, has been demonstrated to activate autophagy in MCF-7 breast cancer cells; nevertheless, the functional role of this autophagy, whether it is protective or cytotoxic and the processes involved were previously ambiguous. Evidence suggests that DOX triggers autophagy as a survival strategy, possibly mediated by reactive oxygen species (ROS). At lower doses (0.05–0.5 μM), DOX mainly induced autophagy, whereas elevated dosages (>1 μM) facilitated apoptosis in both ER-positive MCF-7 and ER-negative MDA-MB-231 cells (p<0.05). The induction of autophagy was verified using acridine orange labeling combined with FACS analysis, with the increase of LC3-II protein and the upregulation of the autophagy regulator Beclin-1. The functional suppression of autophagy using Beclin-1 siRNA led to a twofold increase in DOX-induced apoptosis relative to control siRNA in MCF-7 cells (p<0.05). Subsequent research is investigating whether the suppression of autophagy by Beclin-1 silencing or pharmacological inhibitors such bafilomycin A and hydroxychloroquine amplifies DOX-induced cytotoxicity in several breast cancer cell lines, and if the generation of ROS underlies this impact. The findings suggest that DOX-induced autophagy functions predominantly as a protective mechanism that provides drug resistance, and that pharmacological or genetic suppression of autophagy could be a promising therapeutic strategy to improve chemotherapy effectiveness in breast cancer (136).

In addition to the above studies, the future experiments can focus on understanding the role of tumor metabolism and TME in regulating autophagy-driven DOX resistance. Moreover, hypoxia, starvation and stromal interactions participate to change autophagy, but a number of factors have not been understood in the different cancer subtypes. The metabolic reprogramming toward glycolysis or fatty acid oxidation may intersect with autophagy to provide survival advantages under chemotherapeutic stress. Similarly, immune modulation in the TME such as suppression of T-cell activity or recruitment of autophagy-regulated myeloid-derived suppressor cells could affect tumor persistence despite DOX exposure. Further work could therefore evaluate whether interventions that remodel the metabolic and immune landscape (metabolic inhibitors, immune checkpoint blockade, or TME-targeted nanocarriers) synergize with autophagy inhibition to overcome chemoresistance. Additionally, mapping tissue- or subtype-specific variations in autophagy pathways using multi-omics and patient-derived xenograft models would provide better insight into the heterogeneity of DOX responses in different cancers. Another important direction involves broadening the scope of molecular regulators implicated in DOX resistance beyond the well-characterized circRNAs, lncRNAs, and miRNAs. Novel concepts of regulation such as epigenetic modifications (DNA methylation, histone acetylation), post-translational modifications of autophagy proteins (ubiquitination, phosphorylation), and interactions with other stress-response pathways such as ferroptosis or ER stress remain underexplored but may prove critical in determining whether autophagy serves as a pro-survival or pro-death mechanism. Likewise, receptor-mediated mitophagy regulators (BNIP3, NIX, FUNDC1) and lipid raft–associated signaling add further complexity, potentially linking autophagy with processes including membrane trafficking, lysosomal function, and vesicle dynamics. Importantly, biomarkers such as HMGB1, ATP6AP1, or CEMM components could be tested in clinical cohorts to validate their predictive value for DOX resistance and prognosis. Future studies integrating systems biology approaches, single-cell analysis, and autophagy biosensors could unravel these intricate networks, thereby paving the way for personalized therapeutic strategies that combine DOX with autophagy modulators or complementary targeted therapies.

**Figure 2:**
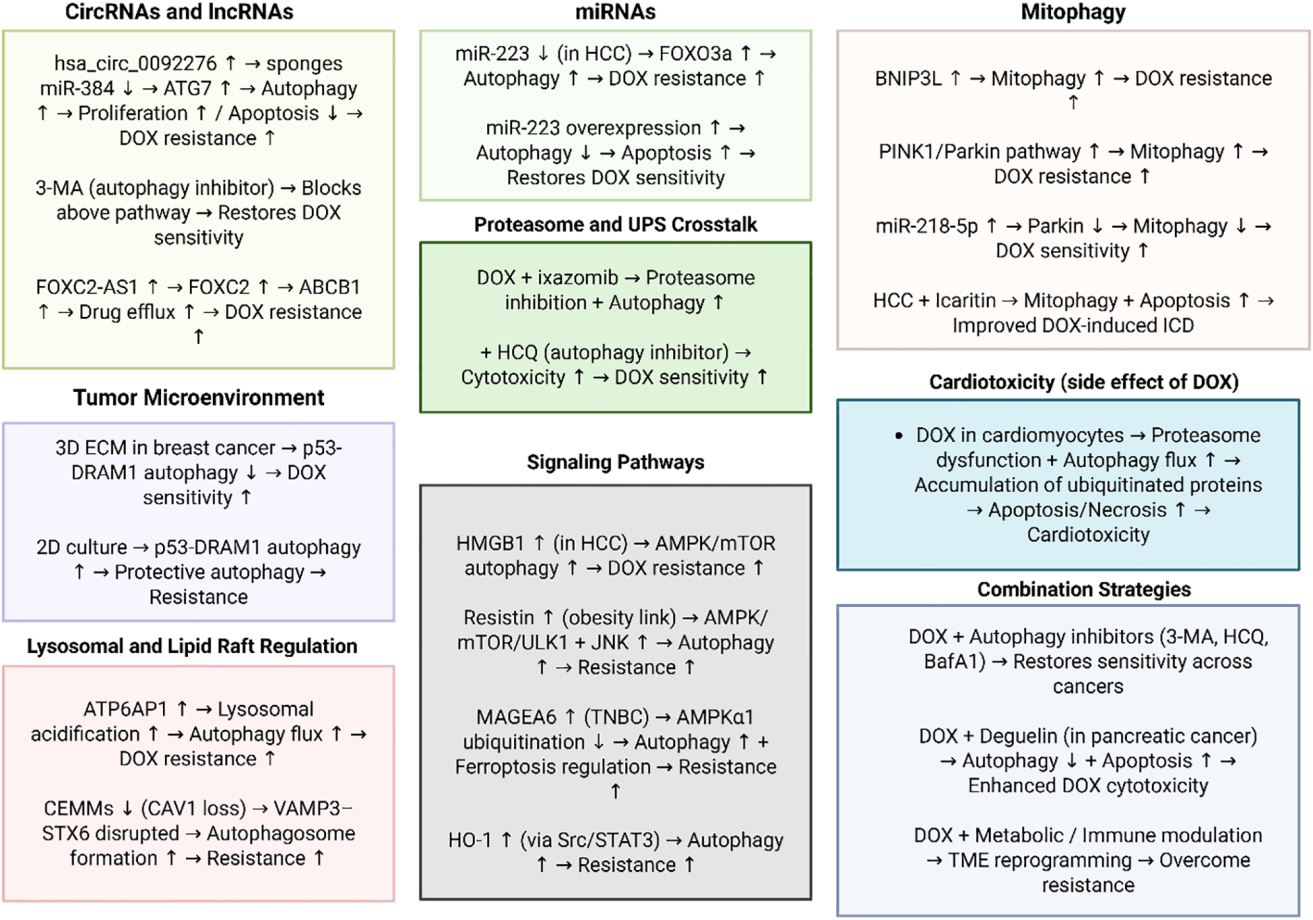
The function of autophagy in DOX resistance. This figure illustrates the multifaceted mechanisms by which autophagy contributes to DOX resistance across different cancer types. Non-coding RNAs, including circRNAs (hsa_circ_0092276), miRNAs (miR-223, miR-218-5p), and lncRNAs (e.g., FOXC2-AS1), regulate autophagy-related genes and drug efflux transporters to promote survival under DOX treatment. Distinct pathways such as PINK1/Parkin- and BNIP3L-mediated mitophagy, HMGB1/AMPK/mTOR signaling, Src/STAT3/HO-1 activation, and lysosomal acidification via ATP6AP1 further sustain adaptive responses to chemotherapy. The tumor microenvironment, lipid raft integrity, and proteasome–autophagy crosstalk add additional layers of context-dependent regulation. Collectively, these mechanisms highlight autophagy as a critical pro-survival process underlying DOX resistance, while pharmacological or genetic inhibition of autophagy restores chemosensitivity, underscoring its therapeutic potential as a target to overcome resistance. (Biorender.com).

Although there have been significant focuses on the regulation of autophagy in DOX resistance, the studies have not considered the autophagy function in the different stages of tumor progression. The comprehensive studies are required to show the status of autophagy in the different stages of tumorigenesis and then, the response to DOX chemotherapy could be evaluated. In addition, the response of the different subtypes of cancers to the DOX chemotherapy considering autophagy status should be evaluated. **Figure 2** further highlights the role of autophagy in DOX resistance.

## 5 Therapeutic implications and targeting strategies

### 5.1 Pharmacological compounds

Recent research emphasizes several strategies to accelerate the efficacy of DOX in breast and other malignancies by the combination of natural chemicals or repurposed pharmaceuticals affecting autophagy, mitochondrial function, and drug resistance mechanisms. Baicalein was demonstrated to enhance the sensitivity of MDA-MB-231 cells to DOX by facilitating autophagy and mitophagy, resulting in the down-regulation of CDK1 and diminished Drp1-mediated mitochondrial fission, an effect that was counteracted by autophagy suppression (137). Conversely, liensinine functioned as a late-stage autophagy/mitophagy inhibitor by inhibiting autophagosome-lysosome fusion through reduced RAB7A recruitment, consequently facilitating DOX-induced apoptosis via increased mitochondrial fission, in both *in vitro* and *in vivo* xenograft models (138). Finally, the antidiabetic drug known as canagliflozin enhanced DOX cytotoxicity by diminishing P-glycoprotein levels and intracellular ATP, thereby facilitating drug absorption, while concurrently inhibiting DOX-induced autophagy via ULK1 phosphorylation, further enhancing therapeutic efficacy in resistant cancer models and xenografts (139).

Colorectal cancer ranks as the third foremost cause of cancer mortality globally for both genders. The conventional therapies encompass surgery, chemotherapy, and targeted therapy; yet, extended exposure to chemical agents frequently leads to the toxicity and drug resistance. A treatment regimen that integrates DOX, metformin, and sodium oxamate, known as triple therapy (Tt), significantly decreased proliferation in colorectal cancer-derived cells by inhibiting the mTOR/AKT pathway while enhancing apoptosis and autophagy in contrast to DOX alone. Western blot examination revealed that several autophagy-related proteins, including as ULK1, ATG4, and LC3 II, were elevated by Tt, with ULK1 demonstrating a gradual increase in expression during the therapy. This demonstrated a post-transcriptional regulation mechanism involving microRNAs, particularly mir-26a, which is known to be upregulated in advanced colorectal cancer and is anticipated to target ULK1. *In vitro* tests demonstrated that mir-26a overexpression decreased ULK1 mRNA and protein levels, while Tt therapy restored ULK1 expression by downregulating mir-26a. Given that Tt inhibited mir-26a expression, a function in transcriptional control was postulated. The examination of the mir-26a promoter identified two binding sites for the transcription factor HIF-1α. The stabilization of HIF-1α in hypoxic circumstances resulted in the overexpression of mir-26a and a reduction in ULK1, with immunoprecipitation verifying the binding of HIF-1α to the mir-26a promoter. Tt significantly reduced HIF-1α levels and restored ULK1 mRNA expression. These findings reveal a regulatory mechanism wherein HIF-1α stimulation induces mir-26a transcription, thus inhibiting ULK1, while triple treatment mitigates this route to promote autophagy and apoptosis in colorectal cancer (140).

Sulforaphane is considered as a natural inhibitor of HDAC and has been evaluated for its efficacy to reduce proliferation of breast cancer and enhance therapeutic efficacy of DOX. Sulforaphane has been shown to decrease proliferation and stimulate apoptosis along with autophagy induction in breast cancer. In addition, sulforaphane is able to stimulate autophagy through HDAC4 downregulation, further enhancing PTEN acetylation and membrane translocation. Assessment using the Chou-Talalay model indicated that sulforaphane in conjunction with DOX has a synergistic effect on growth inhibition. *In vivo* experiments with MDA-MB-231 xenografts demonstrated that the combined therapy caused a more potent antitumor impact than each drug alone. The data indicate that targeting HDAC6 to stimulate autophagy, in conjunction with chemotherapy, may constitute a potential treatment approach for breast cancer (141).

The liver CSCs have been highlighted as vital factors in the induction of DOX resistance in HCC. In order to address this issue, CD133 aptamer has been used to provide targeted delivery of DOX to the liver CSCs with the aim of addressing chemoresistance. A combination of autophagy inhibition and CD133 aptamer-DOX conjugates is evaluated to show how this combination is abele to provide cancer treatment and regulate autophagy. Binding kinetics and thermodynamics, autophagy induction, apoptosis, and self-renewal were evaluated via isothermal titration calorimetry, Western blotting, annexin V assays, and tumorsphere formation assays, whereas aptamer-cell interaction and intracellular drug accumulation were quantified using flow cytometry. Targeted administration by CD133 aptamers significantly elevated intracellular DOX concentrations in liver CSCs. Furthermore, the combination of aptamer-DOX conjugates with autophagy suppression resulted in almost a tenfold increase in the eradication of liver CSCs compared to free DOX *in vitro*. The findings indicate that combining CSC-targeted DOX administration with autophagy suppression may yield a more efficacious treatment approach for HCC (142).

Chalcone flavokawain B (FKB) is recognized for its chemopreventive and anticancer attributes, whereas DOX is a commonly employed DNA-intercalating chemotherapeutic drug. The synergistic effects of FKB (1.25–5 µg/mL) and DOX (0.5 µg/mL) were examined in human gastric cancer (AGS) cells to assess their roles in modulating apoptosis and autophagy, as well as the underlying processes, both *in vitro* and *in vivo*. Cell viability was tested using the MTT assay, protein expression associated with apoptosis and autophagy was examined using Western blot, and synergy was determined with the Chou-Talalay combination index (CI) approach. The *in vivo* effectiveness was also assessed in BALB/c mice. The results indicated that modest dosages of FKB in conjunction with DOX more effectively inhibited AGS cell proliferation than each therapy alone. The combination enhanced DNA fragmentation, apoptotic cell death, and the activation of mitochondrial and death receptor pathways triggered by DOX. It also elevated LC3-II accumulation, p62/SQSTM1 expression, and the production of acidic vesicular organelles, so verifying the activation of autophagy. The modified ratios of Bax/Bcl-2 and Beclin-1/Bcl-2 further suggested simultaneous activation of apoptosis and autophagy. The suppression of apoptosis by Z-VAD-FMK reduced the downregulation of LC3-II/AVO, whereas autophagy suppression via 3-methyladenine or chloroquine attenuated apoptosis by decreasing DNA fragmentation and caspase-3 activation. The activation of ERK/JNK signaling appears to have a role in both apoptotic and autophagic pathways. The combination treatment induced the production of ROS, and the scavenging of ROS with NAC reduced LC3 accumulation, caspase-3 activation, and PARP cleavage. *In vivo*, FKB in conjunction with DOX significantly suppressed the development of gastric cancer xenografts relative to individual therapies. The FKB- DOX combination exhibited synergistic antitumor effects by simultaneously inducing apoptosis and autophagy, presenting a viable treatment approach for gastric cancer (143).

Colorectal cancer, breast cancer, pancreatic cancer, and ovarian cancer constitute substantial global health challenges, with chemotherapy, especially DOX, serving as a primary treatment despite its significant toxicity and resistance complications. Recent investigations highlight the possibility of synergizing DOX with natural chemicals or innovative drugs to enhance effectiveness while reducing undesirable effects. The ethanolic extract of Paris polyphylla (EEPP) demonstrated tumor suppression in colorectal cancer DLD-1 cells by activating autophagy, independent of p53 or caspase-3-mediated apoptosis, and enhanced the efficacy of DOX, with the active components identified as pennogenin 3-O-β-chacotrioside and polyphyllin VI (144). Chidamide (CHI), a histone deacetylase inhibitor, diminished breast cancer growth and metastasis, enhanced ULK2-mediated autophagy, and increased cellular sensitivity to DOX-induced apoptosis, indicating its potential application in combating chemoresistance (145). In pancreatic cancer, danthron, sourced from Rheum palmatum, was seen to block autophagy, a protective process activated by DOX, thereby increasing DOX cytotoxicity and facilitating apoptosis. In ovarian cancer, the combination of DOX and withaferin A (WFA) produced synergistic anti-tumor effects, significantly reducing tumor growth and angiogenesis in both 3D and xenograft models via ROS generation, DNA damage, autophagy, and apoptosis induction, while concurrently reducing the necessary DOX dosage (146, 147). Similarly, magnoflorine (Mag), a natural alkaloid, enhanced DOX sensitivity in breast cancer by augmenting DNA damage, inducing cell cycle arrest, promoting apoptosis, and facilitating autophagy through PI3K/AKT/mTOR inhibition and p38 MAPK activation, with *in vivo* models validating significant anti-tumor efficacy and diminished systemic toxicity (148).

When considering the regulation of autophagy by pharmacological compounds in reversing DOX resistance, several additional aspects should be highlighted beyond the direct mechanisms of autophagy induction or inhibition. The temporal dynamics and context-dependency of autophagy regulation are vital. Autophagy can play dual roles, both cytoprotective or cytotoxic, depending on the duration, intensity, and cellular context in which it is activated. Thus, pharmacological interventions should account for the stage-specific impact of autophagy, ensuring that pro-death rather than pro-survival pathways are preferentially engaged. In addition, tumor heterogeneity poses a challenge, as distinct subpopulations of cancer cells (including CSCs, hypoxic regions, or cells with different genetic/epigenetic profiles) may respond differently to autophagy modulation. Hence, precision in pharmacological targeting, potentially through biomarkers such as ULK1, LC3-II, or Beclin-1 expression levels, is essential to avoid unintended cytoprotective autophagy activation. Moreover, systemic toxicity remains a critical concern; modulating autophagy with natural compounds or repurposed drugs may inadvertently affect non-malignant tissues, given that autophagy is vital for normal cellular homeostasis, especially in the heart, liver, and immune system, organs that are already vulnerable to DOX toxicity. Therefore, achieving tumor-specific delivery, perhaps through nanocarriers, aptamers, or conjugate-based drug designs, represents an important aim to enhance therapeutic selectivity and minimize adverse effects. Another aspect to consider is the interaction between autophagy and other resistance-related pathways, such as oxidative stress, DNA repair, epithelial–mesenchymal transition (EMT), and immune evasion. Autophagy intersects with these processes through shared regulators such as AMPK, mTOR, and p53, suggesting that combined modulation strategies may yield stronger therapeutic responses. The coupling autophagy inhibitors with immunotherapies may enhance the presentation of tumor antigens, overcoming immune resistance while improving DOX sensitivity. Additionally, metabolic rewiring in resistant cancer cells, particularly shifts in glycolysis, mitochondrial function, and ATP generation, plays a major role in autophagy regulation. Drugs that perturb metabolic checkpoints such as metformin, oxamate, or canagliflozin, not only inhibit survival-related autophagy but also disrupt the bioenergetic flexibility required for DOX resistance. Beyond cellular mechanisms, TME-related factors such as hypoxia and HIF-1α stabilization mainly dictate autophagy responses and drug sensitivity. Pharmacological agents that normalize tumor vasculature, alleviate hypoxia, or inhibit hypoxia-inducible signaling can indirectly reprogram autophagy toward pro-death roles. Moreover, long-term clinical translation requires consideration of pharmacokinetics, pharmacodynamics, and combination indices to establish optimal drug ratios and dosing schedules. The success of autophagy-modulating therapies will rely not only on their mechanistic efficacy in preclinical models but also on their capacity to achieve sustained, tolerable, and reproducible effects in heterogeneous patient populations. Thus, a multifaceted approach integrating molecular targeting, tumor-specific delivery, metabolic intervention, and TME modulation will be key to harnessing autophagy regulation as a strategy to overcome DOX resistance.

### 5.2 Nanoparticles and delivery systems

Carvacrol, a monoterpenoid flavonoid prevalent in thyme, encounters limitations in commercial uses owing to its physicochemical instability and inadequate water solubility. A carvacrol nanoemulsion (CANE) was manufactured by the ultrasonication process and characterized through dynamic light scattering (DLS), which indicated a negative surface charge of −29.89 mV and an average droplet size of 99.1 nm. CANE demonstrated substantial anticancer efficacy against DOX-resistant A549 lung carcinoma cells (A549DR), promoting apoptosis as shown by elevated levels of Bax, Cytochrome C, and cleaved caspases 3 and 9. CANE induced cellular senescence and cell cycle arrest by diminishing the levels of CDK2, CDK4, CDK6, Cyclin E, and Cyclin D1, while augmenting p21 expression. An inhibitory impact on autophagy was identified, as evidenced by decreased conversion of LC3-I to LC3-II, downregulation of essential autophagy markers ATG5 and ATG7, and overexpression of p62. CANE demonstrates the capacity to cause apoptosis, senescence, cell cycle arrest, and suppress autophagy in A549DR cells, indicating its potential as a therapeutic option for lung cancer therapy (149).

DOX is a commonly utilized primary chemotherapeutic treatment for several malignancies; yet, its clinical usage is constrained by significant adverse effects, necessitating extensive research into more efficient drug delivery systems (DDSs). A new nucleotropic DOX-loaded nanoparticle (DNP) system has been created with a straightforward, cost-effective, and non-biohazardous chemical design that facilitates formulation and administration, presenting potential therapeutic applications. Developed via vortex-assisted complex coacervation, these DNPs demonstrated exceptional efficacy, enhancing the drug’s cell-inhibitory activity by roughly 300-fold across various human cancer cell lines, including osteosarcoma, breast, prostate, and colorectal cancers, while also enhancing therapeutic outcomes against osteosarcoma *in vivo* by tenfold. The nanoparticles demonstrated a slow-release mechanism, targeting the endoplasmic reticulum, compromising mitochondrial integrity, and finally infiltrating the nucleus. Morphological alterations, including cytosolic vacuolization and cytoplasmic budding, both suggestive of autophagy, were also noted. In mice with osteosarcoma, intratumoral delivery of DNPs resulted in reduced tumor sizes and increased necrotic areas relative to controls. These findings highlight the potential of nucleotropic DNPs to significantly boost DOX delivery and anti-cancer efficacy, positioning them as a promising approach for improved cancer treatment (150). Advancements in nanoparticle-based medication delivery have been achieved with lanthanum strontium manganese oxide (LSMO) nanoparticles to accelerate anticancer efficacy via hyperthermia. This method involved conjugating LSMO nanoparticles with folic acid (Fol-LSMO NPs), loading them with DOX (DoxFol-LSMO NPs), and applying them to breast cancer cells. Exposure to hyperthermia at 45 °C resulted in approximately 95% anticancer efficacy, mainly due to increased oxidative stress. Mechanistic studies demonstrated the activation of the intrinsic mitochondria-mediated apoptotic pathway in conjunction with the induction of autophagy. Molecular research highlighted the interaction between apoptosis and autophagy, involving critical regulators such as Beclin1, Bcl2, and Caspase-3, with free ROS serving as mediators. These findings highlight the successful induction of apoptosis and autophagy by the synergistic combination of hyperthermia and DOX release from Fol-LSMO nanoparticles, presenting a novel therapeutic approach for breast cancer (151).

Adenosine triphosphate (ATP)-binding cassette (ABC) transporters play a vital role in multidrug resistance (MDR) in neoplastic cells. In this superfamilyP-gp and multidrug resistance-associated protein 1 (MRP1) are significantly expressed on the membranes of multidrug-resistant cancer cells. The increase of cellular autophagy is considered as a vital step in the development of MDR. A liposomal method was developed to co-encapsulate DOX and chloroquine phosphate (CQ), an autophagy inhibitor, at a weight ratio of 1:2. In tests on drug-resistance reversal, the IC_50_ of DOX/CQ co-encapsulated liposomes in DOX-resistant human breast cancer cells (MCF7/ADR) was 4.7 ± 0.2 μM, indicating a 5.7-fold reduction compared to free DOX (26.9 ± 1.9 μM). In DOX-resistant human acute myelocytic leukemia cells (HL60/ADR), the decrease was significantly higher, demonstrating an IC_50_ of 1.2 ± 0.1 μM, which is 19.5-fold lower than that of free DOX (23.4 ± 2.8 μM). Cellular uptake experiments indicated that DOX accumulation was enhanced in the presence of free CQ, implying potential interactions between CQ and P-glycoprotein/MRP1. Nonetheless, the expression levels of P-gp and MRP1 remained constant. Conversely, the expression of the autophagy marker LC3-II dramatically increased, suggesting that the reversal of MDR was more directly linked to autophagic inhibition than to transporter downregulation. The anti-tumor efficacy was also assessed utilizing an MCF-7/ADR multicellular tumor spheroid model and a transgenic zebrafish model. In all systems, DOX/CQ co-encapsulated liposomes exhibited enhanced anti-tumor efficacy compared to liposomal DOX or DOX administered alone. The encapsulation of CQ with DOX in liposomes significantly increased DOX sensitivity in resistant cancer cells, highlighting a viable approach to address MDR (152).

Inducing tumor cell death via apoptosis and/or ferroptosis pathways has been extensively studied as a strategy for cancer treatment. Nonetheless, treatment efficacy is frequently suppressed by the autophagy-driven self-repair capabilities of tumor cells. An autophagy inhibition-enhanced apoptosis/ferroptosis technique was implemented to tackle this obstacle, facilitating synergistic tumor elimination by DOX-loaded ferric phosphate nanosheets. In tumor tissues, these nanosheets experience selective breakdown due to the TME. The released DOX causes apoptosis by directly destroying DNA and increasing intracellular hydrogen peroxide levels. Simultaneously, Fe³^+^ ions are reduced to Fe²^+^ by a glutathione-mediated redox reaction, facilitated by the overexpression of glutathione in tumor cells. The produced Fe²^+^ then interacts with hydrogen peroxide via the Fenton reaction, yielding extremely reactive hydroxyl radicals. This redox cascade highlights the antioxidant defenses of tumor cells by depleting glutathione and inactivating glutathione peroxidase 4, thereby facilitating lipid peroxidation and initiating ferroptosis. Furthermore, PO_4_³⁻ ions disrupt lysosomal pH equilibrium by producing conjugated acid anions, hence impairing the self-repair capabilities of tumor cells and enhancing treatment effectiveness. This method has significant promise for targeted tumor elimination by integrating autophagy suppression with the activation of apoptosis and ferroptosis, all while ensuring acceptable biocompatibility (153).

To mitigate the detrimental effects of chemotherapy, several nanostructures have been engineered and produced, presenting a possible treatment strategy for breast cancer (BC). A co-delivery nanodrug delivery system (Co-NDDS) was developed utilizing 2,3-dimercaptosuccinic acid (DMSA)-coated Fe_3_O_4_ nanoparticles as the core, enclosed within a chitosan/alginate nanoparticle (CANPs) shell, with DOX and hydroxychloroquine (HCQ) as the encapsulated therapeutics. Smaller nanoparticles loaded with DOX (FeAC-DOX NPs) were integrated into bigger nanoparticles containing HCQ (FeAC-DOX@PC-HCQ NPs) by ionic gelation and solvent evaporation techniques. The Co-NDDS was comprehensively analyzed for physicochemical characteristics, subsequently undergoing *in vitro* assessment of its anticancer efficacy and mechanisms in two breast cancer cell lines, MCF-7 and MDA-MB-231. The findings indicated that the system demonstrated superior physicochemical stability and encapsulation efficiency, facilitating accurate intracellular release affected by pH sensitivity. Furthermore, the nanoparticle technology significantly improved the *in vitro* cytotoxicity of the combination medicines while efficiently inhibiting autophagy in tumor cells. The Co-NDDS exemplifies a promising approach to breast cancer treatment through the integration of co-delivery, controlled release, and autophagy inhibition (154).

CQ, a traditional autophagy inhibitor, has been regarded as a means to enhance tumor susceptibility to chemotherapy drugs. A significant disparity remains between preclinical results and practical implementation, mainly due to the unique pharmacokinetic characteristics of CQ. A pH-responsive, drug-induced self-assembled nanovesicle, designated DC-DIV/C, was designed using the amphiphilic copolymer PPAP to co-deliver DOX hydrochloride (DOX·HCl) and CQ. The physicochemical parameters of DC-DIV/C were methodically analyzed. To evaluate the collaborative interaction and coordinated administration of DOX·HCl and CQ, assessments of cytotoxicity, apoptosis, cellular uptake, and autophagy suppression were conducted in DOX·HCl-resistant cancer cells. The pharmacokinetic properties and anticancer effectiveness were subsequently investigated in rats and nude mice with K562/ADR xenograft tumors. DC-DIV/C effectively co-encapsulated DOX·HCl and CQ at an ideal weight ratio of 1:2. Both *in vitro* and *in vivo* investigations indicated that DC-DIV/C facilitated effective and synchronized distribution of the two medicines throughout blood circulation, cellular absorption, and intracellular release. The suppression of CQ-mediated autophagy increased the chemosensitivity of resistant cells, resulting in a significant decrease in the IC_50_ of DOX·HCl. DC-DIV/C had a significant anticancer impact, achieving a tumor inhibition rate (TIR) of 84.52% in K562/ADR xenograft models. DC-DIV/C serves as a promising and effective nanoplatform for targeted combination therapy, providing significantly enhanced treatment results in drug-resistant malignancies (155).

**Figure 3:**
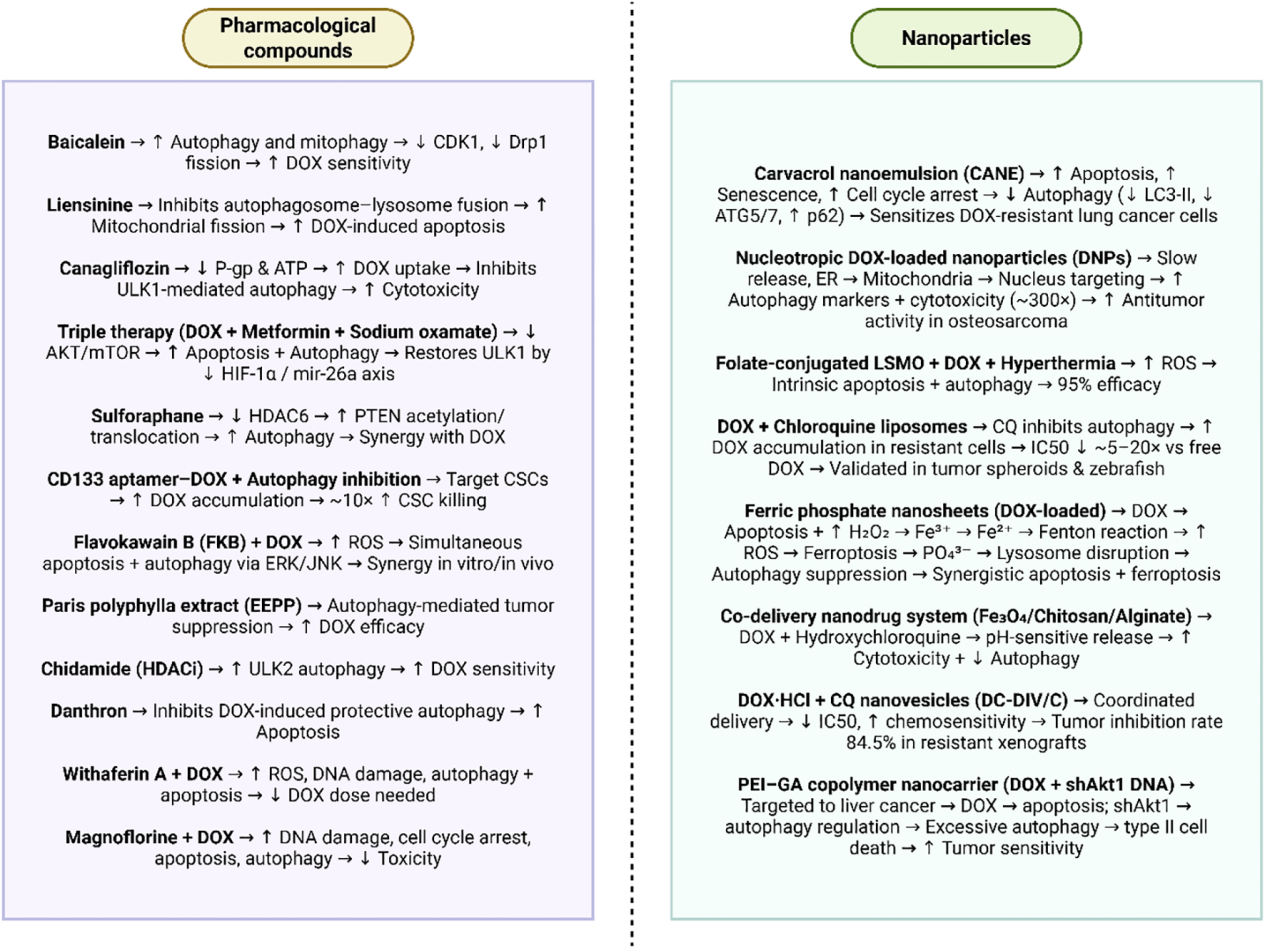
Application of pharmacological compounds and nanoparticles in improving DOX’s activity in cancer therapy. This figure illustrates the therapeutic strategies employed to enhance doxorubicin (DOX) efficacy and overcome drug resistance through two complementary approaches: pharmacological compounds and nanoparticle-based delivery systems. Natural molecules, repurposed drugs, and targeted agents modulate autophagy, mitochondrial function, and drug efflux pathways, either inducing or inhibiting autophagy to shift cancer cells toward apoptosis and heightened DOX sensitivity. Parallel advances in nanotechnology enable precise delivery, co-encapsulation, and tumor microenvironment–responsive release of DOX and autophagy modulators, thereby promoting apoptosis, ferroptosis, and synergistic cytotoxicity while reducing systemic toxicity. Collectively, these strategies highlight a multifaceted approach to reprogram autophagy, disrupt resistance mechanisms, and optimize the therapeutic index of DOX in diverse malignancies. (Biorender.com).

Combination therapy has emerged as a potential approach for treating HCC. A low-toxicity, high-performance nanoparticle system was created via the self-assembly of a poly(ethylenimine)–glycyrrhetinic acid (PEI–GA) amphiphilic copolymer, functioning as a flexible dual delivery platform for pharmaceuticals and genetic material. PEI–GA was produced by the chemical conjugation of hydrophobic GA molecules to the hydrophilic PEI backbone via an acylation process. This nanocarrier effectively encapsulated DOX with a loading capacity of around 12% and was able to condense DNA to create PEI–GA/DOX/DNA complexes for concurrent gene and drug delivery. The resultant complexes had an average diameter of 102 ± 19 nm and a zeta potential of 19.6 ± 0.2 mV. These complexes demonstrated targeted transport to liver cancer cells and enabled effective uptake by HepG2 cells. DOX treatment increased apoptosis, whereas the PI3K/Akt/mTOR signaling pathway facilitated the control of autophagy. The administration of PEI–GA/DOX/shAkt1 complexes significantly induced both apoptosis and autophagy, resulting in cellular demise. Furthermore, the stimulation of excessive autophagy initiated type-II programmed cell death and increased the susceptibility of tumor cells to chemotherapy (156). **Figure 3** highlights the application of pharmacological compounds and nanoparticles in improving DOX’s activity in cancer therapy.

In addition, there are other aspects of nanoparticle-based delivery of DOX for cancer therapy that should be considered include the pharmacokinetics and biodistribution of these nanosystems. Many nanoparticle formulations succeed *in vitro* but fail *in vivo* due to rapid clearance by the reticuloendothelial system (RES), opsonization by serum proteins, or off-target accumulation in healthy tissues. Thus, surface modification strategies such as PEGylation, biomimetic coatings, or ligand conjugation are essential to prolong circulation time, evade immune detection, and improve tumor-specific delivery. Moreover, TME heterogeneity including variations in pH, hypoxia, enzymatic activity, and vascular permeability directly affects nanoparticle penetration and drug release. Responsive or “smart” nanoparticle systems that adapt to these conditions (pH-sensitive, redox-responsive, or enzyme-cleavable systems) are vital for ensuring efficient DOX delivery while minimizing systemic toxicity. Long-term biosafety and metabolic fate of nanocarriers also remain underexplored; comprehensive toxicological studies are required to determine how nanoparticles are degraded, excreted, or retained in organs such as the liver, spleen, or kidneys, which directly affects clinical translation. Another important dimension involves personalization, combination therapies, and overcoming adaptive resistance. While co-delivery systems that combine DOX with autophagy inhibitors or ferroptosis inducers are promising, cancer cells demonstrate diverse resistance mechanisms across patients and tumor subtypes. Integrating nanoparticles with genomic, proteomic, or metabolic profiling could enable precision delivery systems tailored to patient-specific tumor biology. Additionally, combinatorial approaches with immunotherapy (immune checkpoint inhibitors) and targeted therapies may further enhance therapeutic efficacy. The immunomodulatory role of nanoparticles should also be considered, since nanocarriers can interact with immune cells, either suppressing or activating anti-tumor responses. Scalable, reproducible, and cost-effective manufacturing methods are another critical factor for clinical application, as many sophisticated nanoparticle formulations encounter barriers in large-scale production. Moreover, regulatory, ethical, and translational aspects including patient safety, quality control, and long-term monitoring are equally vital to bring DOX-loaded nanoparticle therapies from laboratory to clinical reality. **Table 1** further summarizes the role of autophagy in DOX resistance.

**Table 1:** The role of autophagy in DOX resistance.

Cancer Type/Model	Mechanism/Pathway	Autophagy Role	Key Regulators	Effect on DOX Sensitivity	Intervention/Inhibitor	Outcome/Implication	Refs
A549 (lung cancer), U87 (glioma)	WS2/WSe2 nanosheets bind membranes, partly internalized, alter autophagy genes	Induce autophagy, enhance flux	APP ↓, HSP90AA1 ↓, MAPK14 ↑ (WSe2), TNF ↑ (WS2), ATG9B ↑, ATG5 ↑, ATG4C ↑	Increased DOX cytotoxicity (A549: 6–44%, U87: 10–26%)	3-MA reduced sensitization (21% WS2, 13% WSe2)	Low cytotoxicity alone; act as chemosensitizers; enhance apoptosis and DOX efficacy	(157)
MCF-7 (breast cancer), MCF-7/DOX (DOX-resistant)	miR-142-3p targets HMGB1; HMGB1 regulates ATG5, LC3-I/II conversion; autophagy linked to resistance	Autophagy promoted in MCF-7/DOX; inhibition restores sensitivity	HMGB1 ↑, ATG5 ↑, LC3-II ↑ in resistant cells; miR-142-3p ↓; miR-142-3p mimic suppresses HMGB1/autophagy	miR-142-3p overexpression increased DOX-induced apoptosis and reduced viability; HMGB1 overexpression restored resistance	miR-142-3p mimic sensitized MCF-7/DOX; anti-miR-142-3p conferred resistance; si-HMGB1 reversed resistance	miR-142-3p enhances DOX efficacy *in vitro* and *in vivo* by inhibiting HMGB1-driven autophagy; potential therapeutic strategy	(158)
A2780 (cisplatin-sensitive), A2780cp (cisplatin-resistant) ovarian cancer	Nrf2 pathway activation in resistant cells; regulates autophagy via Atg3, Atg5, Atg12, beclin 1, p62	Autophagy elevated in resistant cells, supports survival	Nrf2 ↑, Keap1 ↑, HO-1 ↑, NQO1 ↑, Atg3 ↑, Atg5 ↑, Atg12 ↑, beclin 1 ↑, p62 ↑	Knockdown of Nrf2 enhanced cisplatin-induced apoptosis (apoptosis ~49% vs 15% in control); reduced viability	Nrf2 siRNA; autophagy inhibition with 3-MA or beclin 1 siRNA increased cisplatin sensitivity	Nrf2 drives both drug resistance and autophagy; blocking Nrf2 or autophagy sensitizes resistant ovarian cancer cells to cisplatin	(159)
TNBC (MDA-MB-231, SUM159PT, resistant sublines R8 & R75)	Epirubicin induces ER stress (PERK, GRP78/BiP) and autophagic flux; resistant cells have higher basal autophagy	Autophagy elevated after epirubicin; resistant lines maintain higher basal autophagic flux; promotes survival	LC3B-II ↑, ATG5 ↑, ATG7 ↑, ER stress proteins (EIF2AK3/PERK, HSPA5/GRP78) ↑	Resistant lines (R8, R75) showed 8–147× higher IC50; autophagy inhibition restored sensitivity; epirubicin + HCQ significantly reduced tumor growth *in vivo*	ATG5/7 siRNA, chloroquine (CQ), hydroxychloroquine (HCQ) combined with epirubicin; Bafilomycin A1 used for flux assay	Genetic or pharmacologic autophagy inhibition sensitized both sensitive and resistant TNBC cells; combination (epirubicin + HCQ/CQ) most effective *in vitro* & *in vivo*; safe toxicity profile	(160)
Osteosarcoma (U2OS, SaoS2, MG63; OSCs CD133+/CD44+)	EGCG synergizes with DOX by inhibiting autophagy; targets lncRNA SOX2OT V7; regulates CSCs via Notch3/DLL3	DOX alone induces autophagy (↑LC3 puncta, ↑Atg5, Beclin-1, ↓p62); EGCG suppresses this autophagy; V7 overexpression restores autophagy (↑LC3II/I, ↓p62); CSCs (OSCs) also regulated by V7-mediated autophagy	SOX2OT V7 ↑ in OS tumors and DOX-treated cells; EGCG ↓ V7; Atg5, Beclin-1, Atg7 ↑ with V7; Nanog, OCT4, Sox2, c-Myc, ABCG2 (CSC markers); Notch3 ↑, DLL3 ↑, Hes1/Hey1 ↑ (downregulated by EGCG unless V7 overexpressed)	EGCG+DOX showed synergistic inhibition (CDI<1); autophagy inhibition (3-MA) ↑ DOX effect; autophagy activation (rapamycin) ↓ synergy; V7 overexpression reduced EGCG+DOX effect	EGCG, DOX, 3-MA (autophagy inhibitor), rapamycin (autophagy inducer), V7 overexpression, Notch3 knockdown	EGCG enhances DOX efficacy by inhibiting DOX-induced autophagy partly via SOX2OT V7; EGCG reduces CSC stemness; inhibition partly mediated through Notch3/DLL3; combination EGCG+DOX is effective anti-OS strategy	(161)
Non-small cell lung cancer (A549, Dox-S vs DOX-R; mouse orthotopic model)	HCD induces autophagic cell death; works via Akt/p38/mTOR downregulation, Beclin-1/PI3K-ClassIII–independent pathway; partial apoptotic involvement	HCD causes autophagic cell death (↑LC3-II, ↑p62); apoptosis minor (C-PARP ↑ at high dose); autophagy inhibition (bafilomycin A1) rescues viability → confirms autophagic cell death dominant	Akt ↓, p38 ↓, mTOR ↓ (abolished at high HCD); Beclin-1 ↓; PI3K-ClassIII variable; C-PARP ↑ at higher HCD; LC3-II ↑; p62 ↑	HCD effective in both DOX-S and DOX-R (same IC50 ~20 μM, cytotoxicity at 10 μM); bypasses DOX resistance	HCD alone; HCD + bafilomycin A1 (autophagy inhibitor); compared with DOX	HCD overcomes DOX resistance via autophagy-mediated death; safe *in vivo* (no liver/kidney toxicity, no body weight loss); inhibits lung tumor progression in mice; shows binding to EGFR, ALK, mTOR (modest affinity)	(162)
Breast cancer (MCF-7, T47D, MDA-MB-231, resistant MCF-7/ADM; xenografts; patient tissue)	ADM (doxorubicin/ADM) ↑ TRPC5 → ↑ intracellular Ca²^+^ → activates CaMKKβ/AMPKα → inhibits mTOR/p70S6K → induces protective autophagy	ADM induced ↑LC3-II, ↑LC3 puncta; resistant cells (MCF-7/ADM) had higher basal autophagy; TRPC5 knockdown ↓ LC3-II and puncta; TRPC5 overexpression ↑ autophagy	TRPC5 ↑, LC3-II ↑, Ca²^+^ ↑, p-CaMKKβ ↑, p-AMPKα ↑, p-mTOR ↓, p-p70S6K ↓; LC3 mRNA unchanged	TRPC5 silencing or autophagy inhibition sensitized cells to ADM; TRPC5 overexpression promoted resistance; resistant xenografts showed high LC3	siTRPC5, TRPC5 shRNA (*in vitro* + *in vivo*), Ca²^+^ chelator (BAPTA/AM), CaMKKβ inhibitor (STO-609), AMPK inhibitor (compound C), CQ, 3-MA	TRPC5 induces cytoprotective autophagy under ADM; blocking TRPC5 or CaMKKβ/AMPKα/mTOR signaling sensitizes breast cancer cells to ADM *in vitro*, *in vivo*, and in patient samples; TRPC5 is a potential therapeutic target to overcome ADM resistance	(163)
Hepatocellular carcinoma (Huh7, HepG2 cells; THLE-3 normal liver cells; nude mouse xenografts)	Mn-doped mesoporous silica nanoparticles (HM) loaded with Isorhamnetin (ISO, pro-autophagy) + Doxorubicin (DOX, DNA damage + ROS). Acidic/GSH-rich TME triggers Mn³^+^/^4+^ → Mn²^+^ reduction → Fenton-like reaction → ROS ↑ + drug release.	ISO promoted autophagy flux; HM@ISO@DOX ↑ LC3-II, ↑ p-ULK1, ↑ p-AMPKα, ↓ p62; autophagy contributed to cancer cell death. CQ partially rescued viability → confirms autophagy-dependent death.	LC3-II, p62, ATG5, ULK1, p-AMPKα, ROS, Mn²^+^, DOX-induced H2O2	HM@ISO@DOX showed strongest killing in Huh7/HepG2 vs HM@DOX or HM@ISO alone; low toxicity to normal THLE-3 cells; *in vivo* tumor growth suppression without weight loss; ISO reduced DOX cardiotoxicity (↓ CK-MB).	CQ (chloroquine, autophagy inhibitor) rescued viability; ATG5-KD, ULK1-KD cells showed reduced LC3-II and restored viability; confirmed dependence on AMPK–ULK1–ATG5 autophagy axis.	Synergistic therapy: DOX-induced DNA damage + ISO-induced autophagy + Mn²^+^-mediated CDT → potent tumor suppression, low systemic toxicity, tumor targeting via HA. Promising nanoplatform for HCC therapy.	(164)

↓means downregulated and ↑ means upregulated.

## 6 Clinical translation and future directions

Advanced squamous non-small cell lung cancer (sq-NSCLC) has conventionally been treated with chemotherapy, and more recently, with a combination of chemotherapy and PD-1 immunotherapy. Ibrilatazar (ABTL0812) is an oral compound that specifically triggers cytotoxic autophagy in neoplastic cells and was assessed in the ENDOLUNG study alongside paclitaxel and carboplatin. Patients with stage III/IV squamous non-small cell lung cancer (sq-NSCLC) were administered ibrilatazar at a dosage of 1300 mg thrice daily, in conjunction with paclitaxel (175 mg/m²) and carboplatin (AUC 5) every three weeks for a maximum of eight cycles, followed by maintenance therapy with ibrilatazar until disease progression or intolerable toxicity occurred. In a cohort of 40 enrolled patients (90% male, median age 66, ECOG 0–1), the overall response rate (ORR) was 32.5% (95% CI: 21.3–50.1) in the intention-to-treat population and 52.0% (95% CI: 34.2–65.9) in the efficacy analysis subset of 25 patients, with disease control rates (DCR) of 52.5% (95% CI: 36.1–68.5) and 84.0% (95% CI: 63.9–95.5), respectively. The median progression-free survival was 6.2 months (95% CI: 4.4–8.8) for both cohorts, but the median overall survival was 18.4 months (95% CI: 9.5–NC) and 22.5 months (95% CI: 10.4–NC). The predominant adverse effects included asthenia (62.5%), diarrhea (45.0%), nausea (37.5%), anemia (32.5%), and neutropenia (27.5%). Pharmacokinetic and pharmacodynamic evaluations validated medication efficacy. The data suggest that ibrilatazar, in conjunction with paclitaxel and carboplatin, exhibits promising effectiveness and acceptable safety in sq-NSCLC, warranting continued clinical development (165).

Endocrine therapy is a crucial element of curative treatment for hormone receptor (HR)-positive breast cancer, and its effectiveness may be augmented by the incorporation of metronomic chemotherapy. To elucidate cellular responses to the combined treatment, autophagy-related markers (beclin 1 and LC3) and apoptosis-related markers (TUNEL and M30) were assessed in pre- and post-treatment cancer tissues from the multicenter neoadjuvant trial JBCRG-07, which involved the administration of oral cyclophosphamide plus letrozole to postmenopausal patients with HR-positive breast cancer. Marker alterations were compared with those found following neoadjuvant endocrine treatment alone, concerning clinical response. Metronomic chemoendocrine treatment resulted in elevated levels of autophagy- and apoptosis-related markers, which correlated with clinical response. Conversely, endocrine treatment alone resulted in elevated autophagy-related indicators without any increase in apoptosis-related markers, irrespective of clinical outcomes; moreover, the levels of the apoptosis marker M30 diminished in responders. Therefore, the activation of apoptosis by metronomic chemoendocrine therapy possibly enhances clinical outcomes compared to the endocrine therapy alone, demonstrating a unique cellular response pattern between the two treatment modalities (166).

A randomized controlled experiment assessed the efficacy of autophagy inhibition in enhancing chemotherapy response during the preoperative treatment of pancreatic cancer by administering the autophagy inhibitor HCQ alongside gemcitabine and nab-paclitaxel. Patients with possibly resectable tumors were allocated to receive two cycles of nab-paclitaxel and gemcitabine (PG) either alone or in conjunction with hydroxychloroquine (PGH), followed by surgical resection. The main outcome was histopathologic response, whilst secondary endpoints encompassed CA 19–9 biomarker response and R0 resection rates, with exploratory assessments of autophagy markers, immunological infiltration, and serum cytokines. In the evaluable cohort (34 PGH, 30 PG), the PGH group exhibited significantly enhanced Evans grade histopathologic responses (P = 0.00016) relative to the control group, and the normalization of CA 19–9 correlated with better overall and recurrence-free survival (P < 0.0001). No discrepancies were noted between groups regarding severe adverse events or the administration of chemotherapeutic dosages. Resected tissues from the PGH arm exhibited heightened autophagy inhibition (elevated SQSTM1, P = 0.027) and augmented immune cell tumor infiltration (P = 0.033). Notwithstanding these biological and pathological advancements, overall survival (P = 0.59) and relapse-free survival (P = 0.55) did not exhibit significant differences between treatment groups. The results demonstrate that the incorporation of hydroxychloroquine with preoperative gemcitabine and nab-paclitaxel improves tumor pathological response, serum biomarker enhancement, and immune activation via autophagy inhibition in resectable pancreatic adenocarcinoma (167).

Numerous research examining autophagy-related pathways in cancer treatment indicate both predictive and therapeutic significance. In metastatic colorectal cancer, hypertension associated with bevacizumab may be affected by genetic variants (168); notably, patients with the G allele of the FIP200 rs1129660 SNP exhibited a significantly diminished risk of developing grade 2–3 hypertension in both the TRIBE and FIRE-3 trials, while no correlation was observed in cetuximab-treated controls, indicating a potential predictive biomarker for anti-VEGF toxicity. In addition to biomarkers, early-phase clinical trials have investigated autophagy suppression as a treatment approach. The amalgamation of hydroxychloroquine (HCQ), a clinically accessible autophagy inhibitor, with vorinostat (VOR), an HDAC inhibitor, demonstrated safety at a maximum tolerated dosage of HCQ 600 mg and VOR 400 mg, accompanied by manageable gastrointestinal and hematologic toxicities, modest clinical efficacy in renal cell carcinoma and colorectal cancer, and pharmacodynamic evidence of elevated CDKN1A and CTSD expression in tumor biopsies (169). Likewise, HCQ in conjunction with palbociclib, a CDK4/6 inhibitor, was well-tolerated at doses of HCQ 600 mg bid and palbociclib 200 mg qd, demonstrating promising efficacy in HR+/HER2- breast cancer patients following CDK4/6 inhibitor failure, with a 41.4% objective response rate and a 90% six-month clinical benefit rate (170). Furthermore, the combination of HCQ with temsirolimus, a mTOR inhibitor, was shown to be viable without attaining the maximum tolerable dose of HCQ, resulting in stable disease in the majority of patients and pharmacodynamic evidence of autophagy suppression at elevated HCQ levels (171). These findings underscore the dual function of autophagy in cancer: genetic polymorphisms in autophagy genes may forecast treatment-related toxicities, whereas pharmacologic inhibition of autophagy improves the therapeutic efficacy of targeted agents, necessitating further exploration of biomarker-guided and combination strategies.

Numerous early-phase clinical trials have assessed autophagy regulation in cancer, yielding inconsistent outcomes. A Phase II study demonstrated that intravenous pantoprazole, when administered alongside docetaxel and prednisone to men with metastatic castration-resistant prostate cancer (mCRPC), was safe; however, it did not achieve the established activity threshold, yielding a PSA response rate of 52% (11/21) and a median overall survival of 15.7 months, indicating tolerability but insufficient clinical efficacy to justify further development (172). Conversely, a first-in-human Phase 1/2A study of the synthetic hydroxylated lipid idroxioleic acid (2-OHOA) in glioma and other advanced solid tumors exhibited a positive safety profile, with gastrointestinal effects determining the maximum tolerated dosage at 12,000 mg daily (173). Significantly, 24% of patients with high-grade gliomas experienced clinical improvement, including one extended response lasting over 2.5 years, therefore endorsing its potential as an innovative treatment approach. In contrast, a Phase II trial of hydroxychloroquine (HCQ), an autophagy inhibitor, in metastatic pancreatic cancer demonstrated minimal efficacy, with merely 10% of patients remaining progression-free at 2 months and an absence of consistent pharmacodynamic evidence of autophagy inhibition in patient samples, despite preclinical validation of LC3-II as a biomarker in murine lymphocytes (174). These studies collectively highlight the potential and difficulties of targeting autophagy in cancer treatment. Although several methods, such as 2-OHOA, provide initial indications of sustained efficacy in gliomas, other tactics, such HCQ in pancreatic cancer or the combination of pantoprazole and docetaxel in prostate cancer, seem inadequate as standalone treatments or in combination without more optimization.

One major perspective for controlling autophagy-driven DOX resistance in the clinic is the rational use of autophagy inhibitors in combination therapies. The emerging clinical data across lung, breast, pancreatic, and colorectal cancer trials shows that autophagy has a dual role, sometimes enabling tumor survival under stress, other times contributing to therapy-induced cytotoxicity. In the context of DOX, resistance often arises because tumor cells leverage autophagy as a protective mechanism against DNA damage and oxidative stress. Clinical strategies could therefore follow the model of HCQ–based trials, where inhibition of autophagy restored chemotherapy sensitivity in subsets of patients. For DOX, incorporating clinically validated autophagy blockers such as HCQ, chloroquine, or novel agents including idroxioleic acid (2-OHOA), could enhance efficacy in resistant tumors, provided biomarkers of autophagy flux are monitored. However, inconsistent pharmacodynamic readouts from earlier HCQ studies emphasize the need for robust, real-time biomarkers (LC3, SQSTM1/p62, or circulating exosome signatures) to identify which patients truly depend on autophagy for resistance. In this way, autophagy-targeting agents could be used not broadly, but selectively, in biomarker-enriched populations where DOX -induced autophagy clearly drives resistance.

A second perspective is to explore the context-specific modulation of autophagy rather than its suppression, aligning with lessons from endocrine and chemoendocrine trials in breast cancer. In those studies, metronomic chemotherapy plus letrozole not only boosted autophagy markers but also triggered apoptosis, improving outcomes compared with endocrine therapy alone. This demonstrates that in some therapeutic settings, controlled induction of autophagy can sensitize tumors to death pathways rather than protect them. For DOX, this suggests a promising approach: in cancers where autophagy shifts toward survival, inhibition should dominate; conversely, in tumors where autophagy primes apoptosis, induction strategies might be beneficial. Clinical implementation could involve combination regimens where DOX is paired with drugs that control autophagy toward pro-death signaling (such as mTOR inhibitors, ER stress inducers, or HDAC inhibitors), maximizing tumor kill while reducing resistance. Moreover, overcoming DOX resistance will require an adaptive, biomarker-guided approach, where autophagy modulation is tailored per tumor type and molecular context, mirroring the trend toward precision oncology in other drug-resistance scenarios.

## 7 Autophagy and cancer immunotherapy

### 7.1 Basic science of autophagy and immunotherapy

Myeloid-derived suppressor cells (MDSCs) are immunosuppressive cells that are raised in the majority of cancer patients, with their accumulation and suppressive activity being affected by inflammation. MDSCs facilitate tumor growth by suppressing anti-tumor immunity. It has been already demonstrated that damage-associated molecular pattern (DAMP) molecule known as high-mobility group box protein 1 (HMGB1) facilitates the accumulation and suppressive efficacy of MDSCs and is prevalent in the TME. HMGB1 also promotes tumor cell longevity by inducing autophagy, providing that fact of whether it similarly regulates MDSC survival through this method. The inhibition of autophagy was observed to elevate the quantity of apoptotic MDSCs, indicating that autophagy extends survival and improves viability in these cells. Inhibition of HMGB1 similarly enhanced apoptosis in MDSCs and reduced their autophagy, demonstrating that HMGB1 not only promotes MDSC accumulation but also maintains their survival. Circulating MDSCs have a baseline autophagic phenotype, but tumor-infiltrating MDSCs demonstrate increased autophagy, providing the notion that inflammatory and hypoxic TME facilitate tumor growth by augmenting the immune-suppressive capabilities of MDSCs. These findings demonstrate that, in addition to its established protumor functions, HMGB1 facilitates tumor growth by maintaining MDSC viability through the promotion of a proautophagic state (175).

Early-stage MDSCs (eMDSCs) constitute a recently identified subpopulation of MDSCs in breast cancer tissues and correlate with unfavorable prognosis in affected individuals. In contrast to traditional MDSCs, eMDSCs demonstrate enhanced immunosuppressive capabilities and aggregate within the TME to suppress both innate and adaptive immune responses. These cells are demonstrated to rely on SOCS3 deficiency and are associated with differentiation arrest in the myeloid lineage. Autophagy is a pivotal regulator of myeloid differentiation; nevertheless, the processes via which it affects the formation of eMDSCs are not well understood. To examine this, conditional myeloid SOCS3 knockout mice (SOCS3MyeKO) with EO771 mammary tumors were developed, demonstrating a significant presence of tumor-infiltrating eMDSCs and enhanced immunosuppression both *in vitro* and *in vivo*. eMDSCs derived from SOCS3MyeKO mice demonstrated differentiation arrest within the myeloid lineage, attributable to inadequate autophagy activation via a Wnt/mTOR-dependent pathway. RNA sequencing and microRNA microarray investigations revealed that miR-155-mediated downregulation of C/EBPβ activated Wnt/mTOR signaling, resulting in autophagy repression and inhibiting differentiation in eMDSCs. Furthermore, the suppression of Wnt/mTOR signaling diminished tumor development and the immunosuppressive capabilities of eMDSCs. The data indicate that the inhibition of autophagy, depending on SOCS3 deficiency and mediated by Wnt/mTOR signaling and microRNA control, is vital for enhancing eMDSC survival and affecting the immunosuppressive TME. This method identifies a possible therapeutic target for cancer treatment (176).

Recent findings highlight the critical impact of TME-mediated mechanisms in cancer progression, wherein several stromal and immune cell types are functionally altered to facilitate malignancy. In cervical squamous cell carcinoma (CSCC), PAI-1 derived from cancer-associated fibroblasts (CAFs) induces endothelial–mesenchymal transition (EndoMT) in lymphatic endothelial cells (LECs) via LRP1-mediated activation of AKT/ERK1/2 signaling, thereby promoting neolymphangiogenesis, facilitating tumor cell intravasation and extravasation, enhancing lymphatic metastasis, and correlating with unfavorable prognosis (177). In HCC, tumor-derived soluble substances, including hyaluronan fragments, activate neutrophil autophagy through Erk1/2, p38, and NF-κB signaling pathways, therefore extending neutrophil longevity, maintaining pro-metastatic factors such as MMP9 and oncostatin M, and facilitating tumor growth (178). Concurrent investigations in melanoma and colorectal cancers reveal that the targeting autophagy genes or the pharmacological inhibition of the autophagy regulator PIK3C3/VPS34 can transform immune-cold tumors into immune-inflamed tumors by stimulating the release of chemokines (CCL5, CXCL10), which recruit NK cells and CD^8+^ T cells, thus enhancing the efficacy of immune checkpoint blockade (ICB) therapies (179). Furthermore, the inhibition of the autophagy gene BECN1 or the inhibition of other autophagy-related pathways enhances CCL5 expression via JNK/c-Jun activation, which recruits NK cells to melanomas and enhances patient survival, highlighting the dual function of autophagy as both a tumor-promoting and immune-modulatory mechanism (180). The findings emphasize that tumor-induced cellular reprogramming through fibroblasts, neutrophils, or tumor-intrinsic autophagy, significantly affects metastasis, immune infiltration, and therapeutic response, presenting potential opportunities for prognostic biomarkers and combination therapies aimed at stromal-tumor-immune interactions.

Microsatellite-stable colorectal cancer (MSS-CRC) demonstrates significant resistance to immunotherapy, necessitating the identification of tumor-intrinsic pathways that contribute to this resistance. Elevated tumor expression of the core autophagy gene ATG16L1 is associated with suboptimal clinical response to anti-PD-L1 treatment in KRAS-mutant tumors in the IMblaze370 (NCT02788279) phase III clinical study of atezolizumab for advanced metastatic MSS-CRC. In engineered mouse colon cancer organoids, the absence of Atg16l1 inhibits tumor development in both primary (colon) and metastatic (liver and lung) locations in syngeneic female hosts, mainly due to the increased susceptibility to IFN-γ-mediated immunological pressure. Deficiency of ATG16L1 enhances programmed cell death in colon cancer organoids induced by IFN-γ and TNF, hence elevating vulnerability to host immunity. Furthermore, ATG16L1 loss diminishes tumor stem-like populations *in vivo*, irrespective of adaptive immune responses. These findings highlight autophagy as a clinically significant mechanism of immune evasion and tumor persistence in MSS-CRC, reinforcing the justification for targeting autophagy to improve immunotherapy results (181).

The efficacy of anti-PD-1 treatment is primarily limited by the diminished frequency of T-cell immune responses and the capacity of tumor cells to prevent immune detection. ATG7, a crucial regulator of autophagy, has been associated with cancer; however, its function in the response to immune checkpoint blockade (ICB) in high microsatellite instability (MSI-H)/mismatch repair-deficient (dMMR) CRC is not yet elucidated. Patients from The Cancer Genome Atlas (TCGA) COAD/READ cohorts were examined to investigate the molecular pathways linked to ATG7. Experimental methodologies including colony formation, cell viability studies, quantitative reverse transcription polymerase chain reaction (qRT-PCR), western blotting, immunofluorescence, flow cytometry, enzyme-linked immunosorbent assay (ELISA), immunohistochemistry, and *in vivo* tumorigenicity assessments. ATG7 was recognized as a pivotal element in MSI-H colorectal carcinoma. Silencing of ATG7 reduced tumor proliferation and augmented the infiltration of CD^8^+^^ T effector cells *in vivo*. Inhibition of ATG7 reinstated surface major histocompatibility complex I (MHC-I) expression, therefore elevating antigen presentation and T-cell–mediated anti-tumor efficacy via activation of the ROS/NF-κB pathway. Moreover, the suppression of ATG7 decreased cholesterol accumulation, hence enhancing anti-tumor immune responses. The suppression of ATG7 combined with statin drug enhanced the effectiveness of anti-PD-1 therapy in MSI-H colorectal cancer. Patients with elevated levels of both ATG7 and 3-hydroxy-3-methylglutaryl coenzyme A reductase (HMGCR) demonstrated a poor prognosis than those with low levels of both markers. Inhibition of ATG7 elevates MHC-I expression, stimulates immunological responses, and reduces cholesterol buildup. These findings highlight the therapeutic potential of targeting ATG7 and demonstrate that statins may enhance sensitivity to immune checkpoint inhibitors in MSI-H CRC (182).

The studies highlight the diverse roles of autophagy in cancer progression, immune evasion, and immunotherapy response. In ovarian cancer, C-MYC was shown to directly bind and downregulate NCOA4 mRNA, thereby inhibiting ferritin autophagy and ferroptosis, reducing ROS production, blocking mitophagy, and finally promoting tumor proliferation, invasion, immune evasion, and tumorigenesis through suppression of HMGB1 release (183). In endometrial cancer, autophagy was found to inhibit the expression of NLRC5, a key MHC-I transactivator, via direct interaction of LC3 with NLRC5, thereby impairing antigen presentation and facilitating immune escape, suggesting that blocking LC3 may restore NLRC5-mediated immune surveillance (184). Meanwhile, in melanoma, targeting the autophagy gene Beclin1 (BECN1) not only inhibited tumor growth but also enhanced NK cell infiltration by inducing CCL5 expression through c-Jun activation, itself driven by impaired PP2A and increased JNK activity (180); importantly, silencing CCL5 abolished NK infiltration and tumor regression. Pharmacological inhibition of autophagy (with chloroquine) or genetic disruption of ATG5 or p62 similarly increased CCL5 expression. Clinically, high CCL5 levels correlated with stronger NK cell presence and improved patient survival. Hence, these findings reveal that autophagy can serve both pro-tumor and anti-tumor roles depending on context, suppressing ferroptosis and antigen presentation in ovarian and endometrial cancers while limiting NK infiltration in melanoma and that manipulating autophagy pathways could provide novel therapeutic avenues for enhancing immunotherapy efficacy across cancer types.

### 7.2 Autophagy induction in cancer immunotherapy

Vaccination holds great potential for revolutionizing disease treatment, yet its widespread clinical application remains limited by challenges such as the absence of safe and efficient delivery systems, poor internalization, and inadequate antigen cross-presentation by dendritic cells (DCs). To address these barriers, a whole cell-encapsulated antitumor vaccine microneedle patch (TCV-DMNs) was developed, designed for transdermal co-delivery of granulocyte-macrophage colony-stimulating factor (GM-CSF) and the autophagy promoter Tat-beclin 1. Upon transdermal administration, GM-CSF released from the microneedles acts as a strong adjuvant to recruit DCs and enhance antigen phagocytosis. Tat-beclin 1 subsequently drives DC maturation and MHC-I-mediated cross-presentation by upregulating autophagy in DCs. Vaccination with TCV-DMNs not only effectively inhibited melanoma growth but also induced regression of established tumors, resulting in relapse-free survival exceeding 40 days. Overall, whole cell-encapsulated microneedle-assisted transdermal vaccination combined with autophagy modulation generates a robust antitumor immune response by enhancing delivery efficiency, improving antigen uptake and cross-presentation, and stimulating T cell activity (185).

Anticancer immunotherapy encounters significant obstacles owing to poor tumor immunogenicity and the existence of an immunosuppressive TME. A liposomal nanodrug was developed to co-encapsulate doxycycline hydrochloride (Doxy) and chlorin e6 (Ce6), facilitating concurrent autophagy suppression and mitochondrial malfunction to improve tumor photo-immunotherapy. Near-infrared laser irradiation of Ce6 generates cytotoxic ROS, initiating robust photodynamic therapy (PDT)-induced immunogenic cell death (ICD) that aids in TME remodeling. Doxy further impairs mitochondrial function, increasing ROS generation and enhancing PDT to promote more effective tumor cell destruction and stronger ICD Doxy inhibits autophagy to increase MHC-I expression on tumor cell surfaces, hence enhancing antigen presentation and cytotoxic T lymphocyte (CTL) identification, which improves tumor immunogenicity. The integration of Ce6-mediated PDT with Doxy-induced autophagy suppression and mitochondrial dysfunction presents a robust therapeutic approach for enhancing cancer immunotherapy (186).

Cancer immunotherapy with ICB constitutes a potential strategy for managing patients with advanced, highly aggressive, and therapy-resistant neoplasms. Nonetheless, only a restricted group of patients attains sustained clinical remission with ICB, since its efficacy predominantly relies on the tumor’s immunological profile, especially the presence of cytotoxic effector immune cells. Genetic targeting of the autophagy-related protein PIK3C3/VPS34 in melanoma and colorectal cancer cells, or the administration of selective inhibitors of PIK3C3/VPS34 kinase activity to tumor-bearing animals, has demonstrated the ability to transform immunological desert tumors into inflammatory, immune-infiltrated tumors. The reprogramming is prompted by the emergence of a proinflammatory profile characterized by the secretion of chemokines CCL5 and CXCL10 in the TME, which aids in the recruitment of NK cells and CD^8+^ T lymphocytes to the tumor. Moreover, the concurrent pharmacological inhibition of PIK3C3/VPS34 with anti-PD-1/PD-L1 therapy amplifies the therapeutic response, demonstrating proof-of-concept for novel clinical trials designed to surmount resistance in cold, IC-unresponsive tumors via dual treatment with PIK3C3/VPS34 inhibitors and ICIs (179).

MDSCs are vital for tumor survival and effectively inhibit anti-tumor immunity, mainly recruited by tumor-derived cytokines such granulocyte-colony stimulating factor (G-CSF) and granulocyte-macrophage colony stimulating factor (GM-CSF). Elevated lactate dehydrogenase A (LDHA) activity in glycolysis is often correlated with increased cytokine levels, which further promotes MDSC recruitment and immunosuppression. A redox-responsive nanoassembly (R-mPDV/PDV/DOX/siL) was developed to elicit robust anti-tumor immunity by integrating LDHA silencing to obstruct cytokine-mediated MDSC recruitment with anthracycline (DOX)-induced ICD to enhance tumor immunogenicity. The nanoassembly is formed by self-assembly from three glutathione (GSH)-responsive polymers, including poly(δ-valerolactone) (PVL) as a hydrophobic component and 3,3′-dithiodipropionic acid (DA) as a cleavable linker to hydrophilic components. DOX is contained within the hydrophobic core, whereas LDHA siRNA (siL) is effectively complexed by cationic PAMAM. The selective binding of the c(RGDfk) (RGD) ligand to integrin αvβ3 enhances targeted cellular uptake and tumor homing. Subsequent to endosomal/lysosomal escape, GSH-mediated cleavage of DA dismantles the nanoassembly, facilitating the rapid release of both DOX and siL, hence achieving effective LDHA silencing. The inhibition of LDHA diminishes the generation of G-CSF and GM-CSF, lowers MDSC recruitment, and enhances anti-tumor immune responses. The simultaneous administration of DOX and siL through the R-mPDV/PDV/DOX/siL nanoassembly demonstrated significant therapeutic effectiveness against 4T1 orthotopic tumors, presenting a promising approach for enhancing immunochemotherapy (187).

Cellular immunotherapies explore to utilize immune cells as agents against cancer, including ex vivo modification of DCs, the initiators of immune responses, demonstrating efficacy in augmenting tumor eradication via enhanced antigen-specific activity. Conversely, the direct stimulation of DCs *in vivo* is both ineffective and exceedingly difficult. A nanoactivator was created to directly activate DCs *in vivo* by enhancing autophagy, thereby facilitating effective antigen presentation and the production of antigen-specific T cells. This method significantly enhances tumor antigen cross-presentation and the ensuing T cell priming. *In vivo* findings indicate that the nanoactivator significantly inhibits tumor proliferation and prolongs lifespan in animal models. *In situ* modification of DCs by autophagy induction is a promising approach for improving antigen presentation and facilitating tumor elimination (188).

Epirubicin (EPI) independently stimulates slightly protective autophagy in remaining tumor cells, providing an immunosuppressive milieu that hastens recurrence and promotes resistance to anti-PD-1/PD-L1 treatments, hence presenting a significant challenge in cancer immunotherapy. Integrating checkpoint drugs that target the PD-1/PD-L1 pathway with methods that increase autophagy is a viable strategy to avert immune evasion and improve therapeutic recognition. A redox-triggered autophagy-inducing nanoplatform utilizing SA&EA-mediated PD-L1 inhibition was designed to do this. The hyaluronic acid (HA) backbone and arginine section facilitated active tumor targeting, cellular absorption, and profound tissue penetration. In the TME, the PLGLAG peptide was degraded by the overexpressed matrix metalloproteinase-2 (MMP-2), resulting in the release of the PD-L1 inhibitor D-PPA to prevent immune evasion. The potent autophagy inducers STF-62247 and EPI were concurrently produced by the breaking of disulfide bonds sensitive to GSH in tumor cells. The synergistic impact of EPI and STF induced both apoptosis and autophagic cell death, successfully eradicating the bulk of tumor cells. The therapeutic results demonstrated higher effectiveness of the SA&EA nanoplatform in comparison to the single-agent formulations of STF@AHMPP or EPI@AHMPTP. This redox-triggered autophagy-inducing nanoplatform, together with PD-L1 suppression, is an effective approach to enhance chemo-immunotherapy (189).

The anticancer immunity elicited by chemoimmunotherapy is significantly affected by tumor autophagy. In the course of treatment, prompt overactivation of autophagy not only induces increased tumor cell apoptosis but also improves endogenous antigen presentation and the release of immune-stimulating signals from necrotic cells, significantly contributing to therapeutic effectiveness. Nonetheless, attaining accurate and prompt overactivation of autophagy in malignancies continues to pose a significant difficulty. An on-demand autophagy cascade amplification nanoparticle (ASN) was designed to enhance oxaliplatin-based cancer treatment. ASN is formed by the self-assembly of autophagy-responsive C-TFG micelles, which are then complexed with an oxaliplatin prodrug (HA-OXA) via electrostatic interactions. Upon internalization by tumor cells, the HA-OXA shell reacts to the reductive TME, releasing oxaliplatin to induce ICD while moderately promoting autophagy. The exposed C-TFG micelles then react to oxaliplatin-induced autophagy by releasing the powerful autophagy activator STF-62247, therefore efficiently transitioning autophagy into an overactivated state. This method triggers autophagic cell death and improves tumor antigen processing from deceased cells. In CT26 tumor-bearing mice, ASN demonstrates robust immune activation and enhanced anticancer efficacy, owing to its precise on-demand autophagy amplification capabilities (190).

Chemotherapeutic drugs can induce ICD via autophagy activation, therefore enhancing anticancer immunotherapy. Nevertheless, chemotherapy alone often induces only modest, cell-protective autophagy, which is inadequate for attaining robust ICD effectiveness. The inclusion of an autophagy inducer can enhance autophagy, consequently boosting ICD and significantly improving the efficacy of anticancer immunotherapy. To implement this strategy, customized autophagy cascade amplification polymeric nanoparticles, designated STF@AHPPE, were developed to enhance tumor immunotherapy. These nanoparticles are synthesized by conjugating arginine (Arg), poly(ethylene glycol)–polycaprolactone, and EPI to a HA backbone by disulfide bonds, while encapsulating the autophagy inducer STF-62247 (STF) inside. Targeting tumor tissues and internalizing into tumor cells via HA and Arg results in increased glutathione content, which induces disulfide bond breaking, therefore releasing both EPI and STF. This mechanism stimulates vigorous cytotoxic autophagy and strong ICD. In comparison to AHPPE nanoparticles, STF@AHPPE demonstrates enhanced tumor cell cytotoxicity, more significant ICD activity, and enhanced immune activation. This technique signifies a viable strategy for the integration of tumor chemo-immunotherapy with autophagy induction (191).

The efficacy of cancer immunotherapy is frequently challenged by the suppressive tumor immune microenvironment (TIME), wherein antitumor immune cells are suppressed and tumor antigens are susceptible to mutation or deletion. To surmount this obstacle, weakly alkaline layered double hydroxide nanoparticles (LDH NPs) were utilized to neutralize surplus acidity and inhibit tumor cell autophagy for neoadjuvant cancer immunotherapy. The peritumoral delivery of LDH nanoparticles resulted in prolonged and effective acid neutralization within the TIME, suppressed the lysosome-mediated autophagy pathway in neoplastic cells, and increased the infiltration of tumor-associated macrophages and T cells exhibiting anticancer activity. Furthermore, LDH nanoparticles sequestered tumor antigens produced from tumor tissues and significantly inhibited the development of melanoma and colon cancers *in vivo*. The results highlight the potential of LDH nanoparticles as immunomodulators and adjuvants that can reactivate and enhance innate and adaptive immune responses, presenting a viable approach for solid tumor immunotherapy (192).

Recent advancements in nanomedicine have revealed the possibility of integrating autophagy inhibition with photothermal, immunotherapeutic, and sonodynamic treatments to improve tumor therapy effectiveness by modifying the TME. A dendrimer nanomedicine modified with indocyanine green (GIC) and loaded with CQ was formulated, demonstrating remarkable stability, cytocompatibility, and elevated photothermal conversion efficiency to induce apoptosis and ICD upon laser irradiation, while concurrently inhibiting autophagy to enhance DC maturation and CD^4+^/CD^8+^ T cell infiltration (193). CQ improved immunological remodeling by activating NF-κB signaling and repolarizing tumor-associated macrophages (TAMs) to the pro-inflammatory M1 phenotype, further enhanced by ICI with PD-L1 antibody. Polyethylene glycol-conjugated gold nanoparticles (PEG-AuNPs) effectively inhibited the M2 polarization of TAMs both *in vitro* and *in vivo* by causing lysosomal alkalization and membrane permeabilization, which suppressed autophagic flux and activated anticancer immune responses (194). A macrophage-mimetic chlorella-based nanoplatform (MChl-CQ-HP-NP) was developed for sonodynamic therapy, concurrently mitigating hypoxia via photosynthetic oxygen production and suppressing autophagy with chloroquine phosphate while administering hematoporphyrin for improved therapeutic efficacy (195). This strategy facilitated tumor elimination and fostered robust immune memory, hence inhibiting tumor recurrence. These studies highlight the essential function of autophagy inhibition in enhancing nanomaterial-based therapies, specifically through the reprogramming of immune responses and the enhancement of photothermal, immunotherapeutic, and sonodynamic treatments, thereby presenting translational potential for comprehensive cancer therapy.

Autophagy plays a multifaceted role in cancer immunotherapy, providing opportunities for both inhibition and induction strategies depending on the cellular context, and several additional aspects remain underexplored. Beyond tumor-intrinsic effects, autophagy in immune cells such as DCs, TAMs, NK cells, and T cells critically regulates antigen presentation, cytokine release, and cytotoxicity, highlighting the need for cell-type specific modulation that inhibits tumor autophagy while enhancing immune cell function. Autophagy also influences immune checkpoints beyond PD-1/PD-L1, including CTLA-4, TIM-3, LAG-3, and TIGIT, suggesting combination therapies that extend beyond current checkpoint blockade. Its tight connection with tumor metabolism further highlights the potential of co-targeting autophagy with metabolic modulators to reshape the TIME. Additionally, stromal components such as CAFs and endothelial cells utilize autophagy to trigger immune evasion, and targeting these compartments may increase immune infiltration. In adoptive cell therapies such as CAR-T and NK-cell therapies, autophagy modulation could enhance persistence, resistance to exhaustion, and antitumor efficacy. Autophagy also intersects with diverse ICD inducers, including radiotherapy, oncolytic viruses, and nanovaccines, expanding its role in immune priming beyond chemotherapy and PDT. Moreover, tumor cells use autophagy for immune escape by degrading MHC-I molecules or secreting immunosuppressive vesicles, while its involvement in trained immunity opens possibilities for durable innate memory reprogramming. Moreover, the development of reliable biomarkers for patient stratification and precision tools for spatiotemporal control of autophagy such as light-activated or microenvironment-responsive nanoplatforms represents a promising avenue to optimize therapeutic selectivity and overcome current limitations in clinical translation.

## 8 The complex role of autophagy in doxorubicin resistance and immunotherapy: future perspectives

Autophagy represents a paradoxical process in cancer therapy, functioning both as a pro-death and pro-survival mechanism. In the context of DOX, a widely used chemotherapeutic drug, autophagy most often promotes survival and contributes to chemoresistance. Cancer cells exposed to DOX activate autophagy as a protective mechanism, enabling them to recycle cellular components, suppress apoptosis, and adapt metabolically under therapeutic stress. This adaptive response is not uniform across all tumor types; instead, it is context-dependent and shaped by genetic mutations, TME-driven pressures, and non-coding RNA regulation. The mitophagy has been shown to mediate resistance in colorectal and hepatocellular cancer stem cells, while lysosomal remodeling enhances DOX survival pathways in breast cancers. These findings highlight the complexity of autophagy’s role in drug resistance and highlight the necessity of tailoring interventions to cancer type, stage, and microenvironmental conditions. The future of overcoming DOX resistance is in the strategic modulation of autophagy. Preclinical studies demonstrate that autophagy inhibitors such as CQ, 3-methyladenine, and HCQ can resensitize resistant tumors to DOX. Furthermore, combination therapies that integrate autophagy inhibitors with nanoparticles, aptamers, or natural compounds show promise in enhancing drug accumulation within tumor cells while limiting systemic toxicity. Importantly, integrating autophagy modulation with immunotherapy is emerging as a novel approach. DOX itself has immunogenic potential through the induction of ICD, but this is often limited by autophagy-driven survival responses. Strategic induction of autophagy can, paradoxically, enhance immunotherapy in some cases by stimulating antigen presentation and reprogramming the TME, while its inhibition in other contexts may boost cytotoxicity. Thus, the direction of autophagy modulation whether inhibition or selective induction, must be carefully controlled to synergize with immunotherapy. Future perspectives emphasize the need for a precision oncology framework where autophagy modulation is not applied universally but tailored based on biomarkers, tumor subtype, and immune status. Potential biomarkers such as HMGB1, ATP6AP1, and lipid raft-associated proteins could help predict whether autophagy is functioning in a cytoprotective or cytotoxic role. Integration of autophagy-targeting strategies with ICIs, metabolic modulators, or TME–normalizing therapies represents an exciting frontier. Additionally, nanotechnology-based co-delivery systems that combine DOX with autophagy modulators or immune stimulants could enable tumor-specific targeting while minimizing off-target effects. Moreover, single-cell and multi-omics analyses will be critical in mapping autophagy’s dynamic role across different tumors, guiding the rational design of therapies. By embracing the complexity of autophagy and strategically integrating it into chemo-immunotherapeutic regimens, it may be possible to transform DOX from a drug limited by resistance into a cornerstone of more durable and effective cancer treatment.

## 9 Conclusion and remarks

Autophagy has been vital in the regulation of cell homeostasis, survival and developmental stages that is considered as an important intracellular degradation mechanism. However, there are still a number of factors that should be considered, especially the dual function of autophagy in cancer, complicating its regulation and targeting the related molecular pathways. Multiple variants of non-canonical autophagy have been identified; these variants bypass critical complexes and require only a subset of ATG proteins. Further investigation is required into the structure and interactions of ATGs, as well as how autophagy machinery demonstrates cell- or tissue-type specificity. As understanding of autophagy increases, there will be additional potential targets for pharmacological and genetic approaches to disease prevention. Breakthroughs in reversing cancer’s chemoresistance require investigation into three vital areas related to autophagy and DOX resistance.

A vital challenge is in the complex, context-dependent role of autophagy in cancer. In some tumor types, autophagy supprots cells from chemotherapy-induced stress, while in others, it contributes to cell death, making it difficult to establish universal therapeutic strategies. This duality complicates the clinical translation, as inhibiting autophagy may improve chemotherapy sensitivity in certain cancers but risk damaging healthy tissues in others. Furthermore, resistance mechanisms are heterogeneous and affected by tumor genetics, TME-related factors such as hypoxia or microbial interactions, and signaling pathways including AMPK/mTOR and STAT3. Clinical trials are also challenged by the variability of biomarkers including autophagy-related gene expression, lysosomal activity, and non-coding RNA regulation that remain insufficiently standardized for predicting treatment response. Beyond tumor-specific biology, drug resistance is affected by issues such as MRPs, changed mitochondrial function, and the protective role of CSCs, all of which limit the long-term effectiveness of DOX-based therapies. From a perspective standpoint, it also suggests that future clinical studies will possibly emphasize personalized and combinatorial approaches to overcome DOX resistance. Autophagy modulators such as CQ, HCQ, and novel biosensor-based inhibitors are being evaluated alongside standard chemotherapy, immunotherapy, and targeted drugs to enhance therapeutic efficacy. Nanotechnology and drug delivery systems also provide promise, as they allow co-delivery of DOX with autophagy inhibitors or gene-silencing tools, thereby increasing drug accumulation in resistant cells while reducing systemic toxicity. Moreover, the integration of non-coding RNAs and molecular regulators including HO-1, TFEB, or MAGEA6 into therapeutic designs points to a more precision-medicine-oriented future, where resistance pathways are targeted based on tumor subtype and patient-specific biomarkers. Importantly, perspectives also include the need to refine non-invasive autophagy monitoring techniques, such as biosensors, to evaluate therapeutic responses dynamically. Therefore, while autophagy-targeted therapies in combination with DOX face biological and translational hurdles, they represent a promising frontier in cancer treatment, with ongoing efforts expected to shift clinical paradigms toward tailored, less toxic, and more durable therapies.

Across tumors, DOX resistance commonly engages stress-induced autophagy programs, classically AMPK/ULK1 activation with mTOR suppression which sustain survival by recycling substrates and buffering ROS; blocking this AMPK/ULK1 axis or core autophagy components (Beclin-1) can resensitize DOX-resistant cells to apoptosis. Mechanistically, DOX-triggered JNK/Bcl-2–Beclin-1 signaling and selective forms of autophagy such as mitophagy remove damaged mitochondria, lowering lipid-ROS and dampening lipid peroxidation, thereby antagonizing ferroptosis as an escape route from DOX cytotoxicity. In parallel, autophagy crosstalk with the p62/SQSTM1/KEAP1/NRF2 axis stabilizes NRF2, transcriptionally boosting SLC7A11, GPX4 and other antioxidant defenses that raise glutathione capacity and blunt ferroptotic death, another lever of DOX resistance with strong evidence across cancers. Yet autophagy can also tip cells toward ferroptosis: AMPK-phosphorylated Beclin-1 directly binds SLC7A11 to inhibit system Xc⁻, sensitizing cells to ferroptotic lipid damage, while ferritinophagy (NCOA4-mediated) liberates iron to expand the labile pool and accelerate lipid peroxidation. These opposing branches, ferroptosis-suppressive (mitophagy/NRF2-driven redox buffering) versus ferroptosis-promoting (Beclin-1–SLC7A11 blockade, ferritinophagy, lipophagy) help explain why tumors often rely on protective autophagy to withstand DOX, and why rational combinations that inhibit survival autophagy or NRF2 signaling while inducing ferroptosis (targeting SLC7A11/GPX4 or activating the Beclin-1 checkpoint) are compelling strategies to overcome DOX resistance.

## References

[1] Ritz E Küster S Zeier M. Clinical nephrology in 19th century Germany. Am J Nephrol. (1994) 14:443–7. doi: 10.1159/000168762, PMID: 7847483

[2] Paget S. The distribution of secondary growths in cancer of the breast. 1889. Cancer Metastasis Rev. (1989) 8:98–101., PMID: 2673568

[3] Hajdu SI. A note from history: landmarks in history of cancer, part 3. Cancer. (2012) 118:1155–68. doi: 10.1002/cncr.26320, PMID: 21751192

[4] Lipsick J. A history of cancer research: tumor suppressor genes. Cold Spring Harbor Perspect Biol. (2020) 12:a035907. doi: 10.1101/cshperspect.a035907, PMID: 32015099 PMC6996451

[5] Vogelstein B Papadopoulos N Velculescu VE Zhou S Diaz LA Jr Kinzler KW. Cancer genome landscapes. Science. (2013) 339:1546–58. doi: 10.1126/science.1235122, PMID: 23539594 PMC3749880

[6] Maley CC Aktipis A Graham TA Sottoriva A Boddy AM Janiszewska M. Classifying the evolutionary and ecological features of neoplasms. Nat Rev Cancer. (2017) 17:605–19. doi: 10.1038/nrc.2017.69, PMID: 28912577 PMC5811185

[7] Gillies RJ Verduzco D Gatenby RA. Evolutionary dynamics of carcinogenesis and why targeted therapy does not work. Nat Rev Cancer. (2012) 12:487–93. doi: 10.1038/nrc3298, PMID: 22695393 PMC4122506

[8] Hanahan D Weinberg RA. Hallmarks of cancer: the next generation. Cell. (2011) 144:646–74. doi: 10.1016/j.cell.2011.02.013, PMID: 21376230

[9] Somarelli JA. The hallmarks of cancer as ecologically driven phenotypes. Front Ecol evolution. (2021) 9:661583. doi: 10.3389/fevo.2021.661583, PMID: 34703824 PMC8544241

[10] Pienta KJ Hammarlund EU Axelrod R Amend SR Brown JS. Convergent evolution, evolving evolvability, and the origins of lethal cancer. Mol Cancer Res. (2020) 18:801–10. doi: 10.1158/1541-7786.MCR-19-1158, PMID: 32234827 PMC7272288

[11] Niu X You Q Hou K Tian Y Wei P Zhu Y. Autophagy in cancer development, immune evasion, and drug resistance. Drug Resistance Updates. (2025) 78:101170. doi: 10.1016/j.drup.2024.101170, PMID: 39603146

[12] Zhang M Liu C Tu J Tang M Ashrafizadeh M Nabavi N. Advances in cancer immunotherapy: historical perspectives, current developments, and future directions. Mol Cancer. (2025) 24:136. doi: 10.1186/s12943-025-02305-x, PMID: 40336045 PMC12057291

[13] Lu Q Kou D Lou S Ashrafizadeh M Aref AR Canadas I. Nanoparticles in tumor microenvironment remodeling and cancer immunotherapy. J Hematol Oncol. (2024) 17:16. doi: 10.1186/s13045-024-01535-8, PMID: 38566199 PMC10986145

[14] Suwa T Kobayashi M Nam J-M Harada H. Tumor microenvironment and radioresistance. Exp Mol Med. (2021) 53:1029–35. doi: 10.1038/s12276-021-00640-9, PMID: 34135469 PMC8257724

[15] Roy PS Saikia BJ. Cancer and cure: A critical analysis. Indian J Cancer. (2016) 53:441–2. doi: 10.4103/0019-509X.200658, PMID: 28244479

[16] Vasan N Baselga J Hyman DM. A view on drug resistance in cancer. Nature. (2019) 575:299–309. doi: 10.1038/s41586-019-1730-1, PMID: 31723286 PMC8008476

[17] Goodman LS Wintrobe MM Dameshek W Goodman MJ Gilman A McLennan MT. Nitrogen mustard therapy; use of methyl-bis (beta-chloroethyl) amine hydrochloride and tris (beta-chloroethyl) amine hydrochloride for Hodgkin’s disease, lymphosarcoma, leukemia and certain allied and miscellaneous disorders. J Am Med Assoc. (1946) 132:126–32. doi: 10.1001/jama.1946.02870380008004, PMID: 20997191

[18] Farber S Diamond LK. Temporary remissions in acute leukemia in children produced by folic acid antagonist, 4-aminopteroyl-glutamic acid. N Engl J Med. (1948) 238:787–93. doi: 10.1056/NEJM194806032382301, PMID: 18860765

[19] Burke MR Smith AR Zheng G. Overcoming cancer drug resistance utilizing PROTAC technology. Front Cell Dev Biol. (2022) 10:872729. doi: 10.3389/fcell.2022.872729, PMID: 35547806 PMC9083012

[20] Housman G Byler S Heerboth S Lapinska K Longacre M Snyder N. Drug resistance in cancer: an overview. Cancers (Basel). (2014) 6:1769–92. doi: 10.3390/cancers6031769, PMID: 25198391 PMC4190567

[21] Iwai Y Ishida M Tanaka Y Okazaki T Honjo T Minato N. Involvement of PD-L1 on tumor cells in the escape from host immune system and tumor immunotherapy by PD-L1 blockade. Proc Natl Acad Sci U.S.A. (2002) 99:12293–7. doi: 10.1073/pnas.192461099, PMID: 12218188 PMC129438

[22] Leach DR Krummel MF Allison JP. Enhancement of antitumor immunity by CTLA-4 blockade. Science. (1996) 271:1734–6. doi: 10.1126/science.271.5256.1734, PMID: 8596936

[23] Ribas A Wolchok JD. Cancer immunotherapy using checkpoint blockade. Science. (2018) 359:1350–5. doi: 10.1126/science.aar4060, PMID: 29567705 PMC7391259

[24] Nowell PC. The clonal evolution of tumor cell populations. Science. (1976) 194:23–8. doi: 10.1126/science.959840, PMID: 959840

[25] Goldie JH Coldman AJ. The genetic origin of drug resistance in neoplasms: implications for systemic therapy. Cancer Res. (1984) 44:3643–53., PMID: 6744284

[26] Skipper HE Schabel FM Jr. Wilcox WS. Experimental evaluation of potential anticancer agents. xiii. on the criteria and kinetics associated with “curability” of experimental leukemia. Cancer Chemother Rep. (1964) 35:1–111., PMID: 14117037

[27] Yin Z Pascual C Klionsky DJ. Autophagy: machinery and regulation. Microb Cell. (2016) 3:588–96. doi: 10.15698/mic2016.12.546, PMID: 28357331 PMC5348978

[28] Suzuki K Kubota Y Sekito T Ohsumi Y. Hierarchy of Atg proteins in pre-autophagosomal structure organization. Genes Cells. (2007) 12:209–18. doi: 10.1111/j.1365-2443.2007.01050.x, PMID: 17295840

[29] Papinski D Schuschnig M Reiter W Wilhelm L Barnes CA Maiolica A. Early steps in autophagy depend on direct phosphorylation of Atg9 by the Atg1 kinase. Mol Cell. (2014) 53:471–83. doi: 10.1016/j.molcel.2013.12.011, PMID: 24440502 PMC3978657

[30] Kamada Y Funakoshi T Shintani T Nagano K Ohsumi M Ohsumi Y. Tor-mediated induction of autophagy via an Apg1 protein kinase complex. J Cell Biol. (2000) 150:1507–13. doi: 10.1083/jcb.150.6.1507, PMID: 10995454 PMC2150712

[31] Schu PV Takegawa K Fry MJ Stack JH Waterfield MD Emr SD. Phosphatidylinositol 3-kinase encoded by yeast VPS34 gene essential for protein sorting. Science. (1993) 260:88–91. doi: 10.1126/science.8385367, PMID: 8385367

[32] Kihara A Noda T Ishihara N Ohsumi Y. Two distinct Vps34 phosphatidylinositol 3-kinase complexes function in autophagy and carboxypeptidase Y sorting in Saccharomyces cerevisiae. J Cell Biol. (2001) 152:519–30. doi: 10.1083/jcb.152.3.519, PMID: 11157979 PMC2196002

[33] Burman C Ktistakis NT. Regulation of autophagy by phosphatidylinositol 3-phosphate. FEBS Lett. (2010) 584:1302–12. doi: 10.1016/j.febslet.2010.01.011, PMID: 20074568

[34] Obara K Sekito T Niimi K Ohsumi Y. The Atg18-Atg2 complex is recruited to autophagic membranes via phosphatidylinositol 3-phosphate and exerts an essential function. J Biol Chem. (2008) 283:23972–80. doi: 10.1074/jbc.M803180200, PMID: 18586673 PMC3259791

[35] Baba M Takeshige K Baba N Ohsumi Y. Ultrastructural analysis of the autophagic process in yeast: detection of autophagosomes and their characterization. J Cell Biol. (1994) 124:903–13. doi: 10.1083/jcb.124.6.903, PMID: 8132712 PMC2119983

[36] Ohsumi Y. Molecular dissection of autophagy: two ubiquitin-like systems. Nat Rev Mol Cell Biol. (2001) 2:211–6. doi: 10.1038/35056522, PMID: 11265251

[37] Cao Y Cheong H Song H Klionsky DJ. *In vivo* reconstitution of autophagy in Saccharomyces cerevisiae. J Cell Biol. (2008) 182:703–13. doi: 10.1083/jcb.200801035, PMID: 18725539 PMC2518709

[38] Lee YK Lee JA. Role of the mammalian ATG8/LC3 family in autophagy: differential and compensatory roles in the sp*atiotemporal regulation of autophagy* . BMB Rep. (2016) 49:424–30. doi: 10.5483/BMBRep.2016.49.8.081, PMID: 27418283 PMC5070729

[39] Shpilka T Weidberg H Pietrokovski S Elazar Z. Atg8: an autophagy-related ubiquitin-like protein family. Genome Biol. (2011) 12:226. doi: 10.1186/gb-2011-12-7-226, PMID: 21867568 PMC3218822

[40] Noda T Kim J Huang WP Baba M Tokunaga C Ohsumi Y. Apg9p/Cvt7p is an integral membrane protein required for transport vesicle formation in the Cvt and autophagy pathways. J Cell Biol. (2000) 148:465–80. doi: 10.1083/jcb.148.3.465, PMID: 10662773 PMC2174799

[41] Yamamoto H Kakuta S Watanabe TM Kitamura A Sekito T Kondo-Kakuta C. Atg9 vesicles are an important membrane source during early steps of autophagosome formation. J Cell Biol. (2012) 198:219–33. doi: 10.1083/jcb.201202061, PMID: 22826123 PMC3410421

[42] Reggiori F Shintani T Nair U Klionsky DJ. Atg9 cycles between mitochondria and the pre-autophagosomal structure in yeasts. Autophagy. (2005) 1:101–9. doi: 10.4161/auto.1.2.1840, PMID: 16874040 PMC1762033

[43] Kirisako T Ichimura Y Okada H Kabeya Y Mizushima N Yoshimori T. The reversible modification regulates the membrane-binding state of Apg8/Aut7 essential for autophagy and the cytoplasm to vacuole targeting pathway. J Cell Biol. (2000) 151:263–76. doi: 10.1083/jcb.151.2.263, PMID: 11038174 PMC2192639

[44] Nair U Yen WL Mari M Cao Y Xie Z Baba M. A role for Atg8-PE deconjugation in autophagosome biogenesis. Autophagy. (2012) 8:780–93., PMID: 22622160 10.4161/auto.19385 PMC3378420

[45] Epple UD Suriapranata I Eskelinen EL Thumm M. Aut5/Cvt17p, a putative lipase essential for disintegration of autophagic bodies inside the vacuole. J Bacteriol. (2001) 183:5942–55. doi: 10.1128/JB.183.20.5942-5955.2001, PMID: 11566994 PMC99673

[46] Teter SA Eggerton KP Scott SV Kim J Fischer AM Klionsky DJ. Degradation of lipid vesicles in the yeast vacuole requires function of Cvt17, a putative lipase. J Biol Chem. (2001) 276:2083–7. doi: 10.1074/jbc.C000739200, PMID: 11085977 PMC2749705

[47] Yang Z Huang J Geng J Nair U Klionsky DJ. Atg22 recycles amino acids to link the degradative and recycling functions of autophagy. Mol Biol Cell. (2006) 17:5094–104. doi: 10.1091/mbc.e06-06-0479, PMID: 17021250 PMC1679675

[48] Mani J Vallo S Rakel S Antonietti P Gessler F Blaheta R. Chemoresistance is associated with increased cytoprotective autophagy and diminished apoptosis in bladder cancer cells treated with the BH3 mimetic (–)-Gossypol (AT-101). BMC Cancer. (2015) 15:224. doi: 10.1186/s12885-015-1239-4, PMID: 25885284 PMC4409725

[49] Hu F Zhao Y Yu Y Fang J-m Cui R Liu Z-q. Docetaxel-mediated autophagy promotes chemoresistance in castration-resistant prostate cancer cells by inhibiting STAT3. Cancer Lett. (2018) 416:24–30. doi: 10.1016/j.canlet.2017.12.013, PMID: 29246644

[50] Han Z Jing Y Xia Y Zhang S Hou J Meng Y. Mesenchymal stem cells contribute to the chemoresistance of hepatocellular carcinoma cells in inflammatory environment by inducing autophagy. Cell Bioscience. (2014) 4:22. doi: 10.1186/2045-3701-4-22, PMID: 24872873 PMC4036298

[51] Ge J Chen Z Huang J Chen J Yuan W Deng Z. Upregulation of autophagy-related gene-5 (ATG-5) is associated with chemoresistance in human gastric cancer. PLoS One. (2014) 9:e110293. doi: 10.1371/journal.pone.0110293, PMID: 25329677 PMC4201506

[52] Fang L-M Li B Guan J-j Xu H-d Shen G-h Gao Q-g. Transcription factor EB is involved in autophagy-mediated chemoresistance to doxorubicin in human cancer cells. Acta Pharmacologica Sin. (2017) 38:1305–16. doi: 10.1038/aps.2017.25, PMID: 28603284 PMC5589965

[53] Wang L Shang Z Zhou Y Hu X Chen Y Fan Y. Autophagy mediates glucose starvation-induced glioblastoma cell quiescence and chemoresistance through coordinating cell metabolism, cell cycle, and survival. Cell Death Dis. (2018) 9:213. doi: 10.1038/s41419-017-0242-x, PMID: 29434213 PMC5833690

[54] Yu T Guo F Yu Y Sun T Ma D Han J. Fusobacterium nucleatum promotes chemoresistance to colorectal cancer by modulating autophagy. Cell. (2017) 170:548–563.e16. doi: 10.1016/j.cell.2017.07.008, PMID: 28753429 PMC5767127

[55] Kim M Jung J-Y Choi S Lee H Morales LD Koh J-T. GFRA1 promotes cisplatin-induced chemoresistance in osteosarcoma by inducing autophagy. Autophagy. (2017) 13:149–68. doi: 10.1080/15548627.2016.1239676, PMID: 27754745 PMC5240831

[56] Piya S Andreeff M Borthakur G. Targeting autophagy to overcome chemoresistance in acute myleogenous leukemia. Autophagy. (2017) 13:214–5. doi: 10.1080/15548627.2016.1245263, PMID: 27797294 PMC5240828

[57] Wu S Wang X Chen J Chen Y. Autophagy of cancer stem cells is involved with chemoresistance of colon cancer cells. Biochem Biophys Res Commun. (2013) 434:898–903. doi: 10.1016/j.bbrc.2013.04.053, PMID: 23624503

[58] Battista RA Resnati M Facchi C Ruggieri E Cremasco F Paradiso F. Autophagy mediates epithelial cancer chemoresistance by reducing p62/SQSTM1 accumulation. PLoS One. (2018) 13:e0201621. doi: 10.1371/journal.pone.0201621, PMID: 30067838 PMC6070274

[59] Pagotto A Pilotto G Mazzoldi EL Nicoletto MO Frezzini S Pastò A. Autophagy inhibition reduces chemoresistance and tumorigenic potential of human ovarian cancer stem cells. Cell Death Dis. (2017) 8:e2943–3. doi: 10.1038/cddis.2017.327, PMID: 28726781 PMC5550872

[60] Song J Qu Z Guo X Zhao Q Zhao X Gao L. Hypoxia-induced autophagy contributes to the chemoresistance of hepatocellular carcinoma cells. Autophagy. (2009) 5:1131–44. doi: 10.4161/auto.5.8.9996, PMID: 19786832

[61] Al-Malky HS Al Harthi SE Osman AM. Major obstacles to doxorubicin therapy: Cardiotoxicity and drug resistance. J Oncol Pharm Pract. (2020) 26:434–44. doi: 10.1177/1078155219877931, PMID: 31594518

[62] Gewirtz DA. A critical evaluation of the mechanisms of action proposed for the antitumor effects of the anthracycline antibiotics adriamycin and daunorubicin. Biochem Pharmacol. (1999) 57:727–41. doi: 10.1016/S0006-2952(98)00307-4, PMID: 10075079

[63] Sinha BK Mimnaugh EG. Free radicals and anticancer drug resistance: oxygen free radicals in the mechanisms of drug cytotoxicity and resistance by certain tumors. Free Radic Biol Med. (1990) 8:567–81. doi: 10.1016/0891-5849(90)90155-C, PMID: 2113883

[64] Pawłowska J Tarasiuk J Wolf CR Paine MJ Borowski E. Differential ability of cytostatics from anthraquinone group to generate free radicals in three enzymatic systems: NADH dehydrogenase, NADPH cytochrome P450 reductase, and xanthine oxidase. Oncol Res. (2003) 13:245–52. doi: 10.3727/096504003108748294, PMID: 12688675

[65] Fogli S Nieri P Breschi MC. The role of nitric oxide in anthracycline toxicity and prospects for pharmacologic prevention of cardiac damage. FASEB J. (2004) 18:664–75. doi: 10.1096/fj.03-0724rev, PMID: 15054088

[66] Frank NE Cusack BJ Talley TT Walsh GM Olson RD. Comparative effects of doxorubicin and a doxorubicin analog, 13-deoxy, 5-iminodoxorubicin (GPX-150), on human topoisomerase IIβ activity and cardiac function in a chronic rabbit model. Invest New Drugs. (2016) 34:693–700. doi: 10.1007/s10637-016-0388-x, PMID: 27581956

[67] Chen SH Chan NL Hsieh TS. New mechanistic and functional insights into DNA topoisomerases. Annu Rev Biochem. (2013) 82:139–70. doi: 10.1146/annurev-biochem-061809-100002, PMID: 23495937

[68] Roos WP Kaina B. DNA damage-induced cell death: from sp*ecific DNA lesions to the DNA damage response and apoptosis* . Cancer Lett. (2013) 332:237–48. doi: 10.1016/j.canlet.2012.01.007, PMID: 22261329

[69] Lotem J Peled-Kamar M Groner Y Sachs L. Cellular oxidative stress and the control of apoptosis by wild-type p53, cytotoxic compounds, and cytokines. Proc Natl Acad Sci U.S.A. (1996) 93:9166–71., PMID: 8799172 10.1073/pnas.93.17.9166 PMC38613

[70] Paskeh MDA Saebfar H Mahabady MK Orouei S Hushmandi K Entezari M. Overcoming doxorubicin resistance in cancer: siRNA-loaded nanoarchitectures for cancer gene therapy. Life Sci. (2022) 298:120463. doi: 10.1016/j.lfs.2022.120463, PMID: 35259354

[71] Mirzaei S Zarrabi A Hashemi F Zabolian A Saleki H Azami N. Nrf2 signaling pathway in chemoprotection and doxorubicin resistance: potential application in drug discovery. Antioxidants. (2021) 10:349. doi: 10.3390/antiox10030349, PMID: 33652780 PMC7996755

[72] Poh HM Chiou YS Chong QY Chen RM Rangappa KS Ma L. Inhibition of TFF3 enhances sensitivity—and overcomes acquired resistance—to doxorubicin in estrogen receptor-positive mammary carcinoma. Cancers. (2019) 11:1528. doi: 10.3390/cancers11101528, PMID: 31658702 PMC6826976

[73] Rajendran P Ong TH Chen L Li F Shanmugam MK Vali S. Suppression of signal transducer and activator of transcription 3 activation by butein inhibits growth of human hepatocellular carcinoma *in vivo* . Clin Cancer Res. (2011) 17:1425–39. doi: 10.1158/1078-0432.CCR-10-1123, PMID: 21131551

[74] Ashrafizadeh M Zarrabi A Hashemi F Zabolian A Saleki H Bagherian M. Polychemotherapy with curcumin and doxorubicin via biological nanoplatforms: enhancing antitumor activity. Pharmaceutics. (2020) 12:1084. doi: 10.3390/pharmaceutics12111084, PMID: 33187385 PMC7697177

[75] Li Z Li H Liu B Luo J Qin X Gong M. Inhibition of miR-25 attenuates doxorubicin-induced apoptosis, reactive oxygen sp*ecies production and DNA damage by targeting PTEN* . Int J Med Sci. (2020) 17:1415. doi: 10.7150/ijms.41980, PMID: 32624698 PMC7330660

[76] Wang B Xu L Zhang J Cheng X Xu Q Wang J. LncRNA NORAD accelerates the progression and doxorubicin resistance of neuroblastoma through up-regulating HDAC8 via sponging miR-144-3p. Biomedicine Pharmacotherapy. (2020) 129:110268. doi: 10.1016/j.biopha.2020.110268, PMID: 32563146

[77] Xu W-L Shi BJ Li SL Yu FX Guo LN Li M. Targeted inhibition of myeloid-derived suppressor cells in the tumor microenvironment by low-dose doxorubicin to improve immune efficacy in murine neuroblastoma. Chin Med J. (2021) 134:334–43. doi: 10.1097/CM9.0000000000001234, PMID: 33278092 PMC7846436

[78] Morsy MA El-Sheikh AA Ibrahim AR Venugopala KN Kandeel M. In silico and *in vitro* identification of secoisolariciresinol as a re-sensitizer of P-glycoprotein-dependent doxorubicin-resistance NCI/ADR-RES cancer cells. PeerJ. (2020) 8:e9163. doi: 10.7717/peerj.9163, PMID: 32566390 PMC7293189

[79] Raniolo S Unida V Vindigni G Stolfi C Iacovelli F Desideri A. Combined and selective miR-21 silencing and doxorubicin delivery in cancer cells using tailored DNA nanostructures. Cell Death Dis. (2021) 12:7. doi: 10.1038/s41419-020-03339-3, PMID: 33414439 PMC7791072

[80] Boichuk S Bikinieva F Nurgatina I Dunaev P Valeeva E Aukhadieva A. Inhibition of AKT-signaling sensitizes soft tissue sarcomas (STS) and gastrointestinal stromal tumors (GIST) to doxorubicin via targeting of homology-mediated DNA repair. Int J Mol Sci. (2020) 21:8842. doi: 10.3390/ijms21228842, PMID: 33266502 PMC7700672

[81] Chen E Li E Liu H Zhou Y Wen L Wang J. miR-26b enhances the sensitivity of hepatocellular carcinoma to Doxorubicin via USP9X-dependent degradation of p53 and regulation of autophagy. Int J Biol Sci. (2021) 17:781. doi: 10.7150/ijbs.52517, PMID: 33767588 PMC7975695

[82] Kirtonia A Gala K Fernandes SG Pandya G Pandey AK Sethi G. Repurposing of drugs: An attractive pharmacological strategy for cancer therapeutics. In: Seminars in cancer biology, vol. 68. San Diego (CA): Academic Press (2021). p. 258–78., PMID: 32380233 10.1016/j.semcancer.2020.04.006

[83] Li R Zhang B. LINC01116 promotes doxorubicin resistance in osteosarcoma by epigenetically silencing miR-424-5p and inducing epithelial-mesenchymal transition. Front Pharmacol. (2021) 12:632206. doi: 10.3389/fphar.2021.632206, PMID: 33762953 PMC7982720

[84] Vargas JE Puga R Lenz G Trindade C Filippi-Chiela E. Cellular mechanisms triggered by the cotreatment of resveratrol and doxorubicin in breast cancer: a translational *in vitro*–in silico model. Oxid Med Cell Longevity. (2020) 2020:5432651. doi: 10.1155/2020/5432651, PMID: 33204396 PMC7654215

[85] Yan T Zhu S Hui W He J Liu Z Cheng J. Chitosan based pH-responsive polymeric prodrug vector for enhanced tumor targeted co-delivery of doxorubicin and siRNA. Carbohydr polymers. (2020) 250:116781. doi: 10.1016/j.carbpol.2020.116781, PMID: 33049806

[86] Ashrafizadeh M Hushmandi K RahmaniMoghadam E Zarrin V HosseinzadehKashani S Bokaie S. Progress in delivery of siRNA-based therapeutics employing nano-vehicles for treatment of prostate cancer. Bioengineering. (2020) 7:91. doi: 10.3390/bioengineering7030091, PMID: 32784981 PMC7552721

[87] Mirzaei S Gholami MH Hashemi F Zabolian A Hushmandi K Rahmanian V. Employing siRNA tool and its delivery platforms in suppressing cisplatin resistance: approaching to a new era of cancer chemotherapy. Life Sci. (2021) 277:119430. doi: 10.1016/j.lfs.2021.119430, PMID: 33789144

[88] Mirzaei S Mahabady MK Zabolian A Abbaspour A Fallahzadeh P Noori M. Small interfering RNA (siRNA) to target genes and molecular pathways in glioblastoma therapy: Current status with an emphasis on delivery systems. Life Sci. (2021) 275:119368. doi: 10.1016/j.lfs.2021.119368, PMID: 33741417

[89] Delfi M Sartorius R Ashrafizadeh M Sharifi E Zhang Y DeBerardinis P. Self-assembled peptide and protein nanostructures for anti-cancer therapy: Targeted delivery, stimuli-responsive devices and immunotherapy. Nano Today. (2021) 38:101119. doi: 10.1016/j.nantod.2021.101119, PMID: 34267794 PMC8276870

[90] Ashrafizade M Delfi M Hashemi F Zabolian A Saleki H Bagherian M. Biomedical application of chitosan-based nanoscale delivery systems: potential usefulness in siRNA delivery for cancer therapy. Carbohydr. Polym. (2021) 260:10.1016. omid Sharifzadeh S, Hamzehlou S. doi: 10.1016/j.carbpol.2021.117809, PMID: 33712155

[91] Ashrafizaveh S Ashrafizadeh M Zarrabi A Husmandi K Zabolian A Shahinozzaman M. Long non-coding RNAs in the doxorubicin resistance of cancer cells. Cancer Lett. (2021) 508:104–14. doi: 10.1016/j.canlet.2021.03.018, PMID: 33766750

[92] Cao X Hou J An Q Assaraf YG Wang X. Towards the overcoming of anticancer drug resistance mediated by p53 mutations. Drug Resistance Updates. (2020) 49:100671. doi: 10.1016/j.drup.2019.100671, PMID: 31841768

[93] Chan K-T Lung ML. Mutant p53 expression enhances drug resistance in a hepatocellular carcinoma cell line. Cancer chemotherapy Pharmacol. (2004) 53:519–26. doi: 10.1007/s00280-004-0767-4, PMID: 15004724

[94] Xia X Wang Q Ye T Liu Y Liu D Song S. NRF 2/ABCB 1-mediated efflux and PARP 1-mediated dampening of DNA damage contribute to doxorubicin resistance in chronic hypoxic HepG2 cells. Fundam Clin Pharmacol. (2020) 34:41–50. doi: 10.1111/fcp.12505, PMID: 31420991

[95] Dai X Wang L Deivasigamni A Looi CY Karthikeyan C Trivedi P. A novel benzimidazole derivative, MBIC inhibits tumor growth and promotes apoptosis via activation of ROS-dependent JNK signaling pathway in hepatocellular carcinoma. Oncotarget. (2017) 8:12831. doi: 10.18632/oncotarget.14606, PMID: 28086233 PMC5355059

[96] Kirtonia A Sethi G Garg M. The multifaceted role of reactive oxygen sp*ecies in tumorigenesis* . Cell Mol Life Sci. (2020) 77:4459–83. doi: 10.1007/s00018-020-03536-5, PMID: 32358622 PMC11105050

[97] Bock FJ Tait SW. Mitochondria as multifaceted regulators of cell death. Nat Rev Mol Cell Biol. (2020) 21:85–100. doi: 10.1038/s41580-019-0173-8, PMID: 31636403

[98] Zhu Y Xu J Hu W Wang F Zhou Y Xu W. TFAM depletion overcomes hepatocellular carcinoma resistance to doxorubicin and sorafenib through AMPK activation and mitochondrial dysfunction. Gene. (2020) 753:144807. doi: 10.1016/j.gene.2020.144807, PMID: 32461017

[99] Xie X Hu Y Xu L Fu Y Tu J Zhao H. The role of miR-125b-mitochondria-caspase-3 pathway in doxorubicin resistance and therapy in human breast cancer. Tumor Biol. (2015) 36:7185–94. doi: 10.1007/s13277-015-3438-7, PMID: 25894378

[100] Shin DH Choi Y-J Park J-W. SIRT1 and AMPK mediate hypoxia-induced resistance of non–small cell lung cancers to cisplatin and doxorubicin. Cancer Res. (2014) 74:298–308. doi: 10.1158/0008-5472.CAN-13-2620, PMID: 24240701

[101] Taymaz-Nikerel H Karabekmez ME Eraslan S Kırdar B. Doxorubicin induces an extensive transcriptional and metabolic rewiring in yeast cells. Sci Rep. (2018) 8:13672. doi: 10.1038/s41598-018-31939-9, PMID: 30209405 PMC6135803

[102] Bramwell VH Morris D Ernst DS Hings I Blackstein M Venner PM. Safety and efficacy of the multidrug-resistance inhibitor biricodar (VX-710) with concurrent doxorubicin in patients with anthracycline-resistant advanced soft tissue sarcoma. Clin Cancer Res. (2002) 8:383–93., PMID: 11839653

[103] Le Cesne A Vassal G Farace F Spielmann M LeChevalier T Angevin E. Combination interleukin-2 and doxorubicin in advanced adult solid tumors: circumvention of doxorubicin resistance in soft-tissue sarcoma? Philadelphia (PA): Lippincott Williams & Wilkins (LWW). (1999) p. 268–77. 10.1097/00002371-199905000-00010 10335487

[104] Advani R Fisher GA Lum BL Hausdorff J Halsey J Litchman M. A phase I trial of doxorubicin, paclitaxel, and valspodar (PSC 833), a modulator of multidrug resistance. Clin Cancer Res. (2001) 7:1221–9., PMID: 11350887

[105] Fracasso P Blum K Ma M Tan B Wright L Goodner S. Phase I study of pegylated liposomal doxorubicin and the multidrug-resistance modulator, valspodar. Br J Cancer. (2005) 93:46–53. doi: 10.1038/sj.bjc.6602653, PMID: 15942626 PMC2361488

[106] Shang J Chen W-M Liu S Wang Z-H Wei T-N Chen Z-Z. CircPAN3 contributes to drug resistance in acute myeloid leukemia through regulation of autophagy. Leukemia Res. (2019) 85:106198. doi: 10.1016/j.leukres.2019.106198, PMID: 31401408

[107] Du F Yu L Wu Y Wang S Yao J Zheng X. miR-137 alleviates doxorubicin resistance in breast cancer through inhibition of epithelial-mesenchymal transition by targeting DUSP4. Cell Death Dis. (2019) 10:922. doi: 10.1038/s41419-019-2164-2, PMID: 31801953 PMC6892819

[108] Yan L Ding B Liu H Zhang Y Zeng J Hu J. Inhibition of SMYD2 suppresses tumor progression by down-regulating microRNA-125b and attenuates multi-drug resistance in renal cell carcinoma. Theranostics. (2019) 9:8377. doi: 10.7150/thno.37628, PMID: 31754403 PMC6857066

[109] Wang Q Liang D Shen P Yu Y Yan Y You W. Hsa_circ_0092276 promotes doxorubicin resistance in breast cancer cells by regulating autophagy via miR-348/ATG7 axis. Trans Oncol. (2021) 14:101045. doi: 10.1016/j.tranon.2021.101045, PMID: 34023560 PMC8163983

[110] Zhang C-L Zhu K-P Ma X-L. Antisense lncRNA FOXC2-AS1 promotes doxorubicin resistance in osteosarcoma by increasing the expression of FOXC2. Cancer Lett. (2017) 396:66–75. doi: 10.1016/j.canlet.2017.03.018, PMID: 28323030

[111] Zhou Y Chen E Tang Y Mao J Shen J Zheng X. miR-223 overexpression inhibits doxorubicin-induced autophagy by targeting FOXO3a and reverses chemoresistance in hepatocellular carcinoma cells. Cell Death Dis. (2019) 10:843. doi: 10.1038/s41419-019-2053-8, PMID: 31695022 PMC6834650

[112] Yan C Luo L Guo C-Y Goto S Urata Y Shao J-H. Doxorubicin-induced mitophagy contributes to drug resistance in cancer stem cells from HCT8 human colorectal cancer cells. Cancer Lett. (2017) 388:34–42. doi: 10.1016/j.canlet.2016.11.018, PMID: 27913197

[113] Gomes LR Vessoni AT Menck CFM. Three-dimensional microenvironment confers enhanced sensitivity to doxorubicin by reducing p53-dependent induction of autophagy. Oncogene. (2015) 34:5329–40. doi: 10.1038/onc.2014.461, PMID: 25619836

[114] Loh JS Rahim NA Tor YS Foo JB. Simultaneous proteasome and autophagy inhibition synergistically enhances cytotoxicity of doxorubicin in breast cancer cells. Cell Biochem Funct. (2022) 40:403–16. doi: 10.1002/cbf.3704, PMID: 35485606

[115] Naso FD Bruqi K Manzini V Chiurchiù V D’Onofrio M Arisi I. miR-218-5p and doxorubicin combination enhances anticancer activity in breast cancer cells through Parkin-dependent mitophagy inhibition. Cell Death Discov. (2024) 10:149. doi: 10.1038/s41420-024-01914-7, PMID: 38514650 PMC10957887

[116] Yu Z Guo J Hu M Gao Y Huang L. Icaritin exacerbates mitophagy and synergizes with doxorubicin to induce immunogenic cell death in hepatocellular carcinoma. ACS Nano. (2020) 14:4816–28. doi: 10.1021/acsnano.0c00708, PMID: 32188241

[117] Ma M Lin XH Liu HH Zhang R Chen RX. Suppression of DRP1−mediated mitophagy increases the apoptosis of hepatocellular carcinoma cells in the setting of chemotherapy. Oncol Rep. (2020) 43:1010–8. doi: 10.3892/or.2020.7476, PMID: 32020220

[118] Dimitrakis P Romay-Ogando MI Timolati F Suter TM Zuppinger C. Effects of doxorubicin cancer therapy on autophagy and the ubiquitin-proteasome system in long-term cultured adult rat cardiomyocytes. Cell Tissue Res. (2012) 350:361–72. doi: 10.1007/s00441-012-1475-8, PMID: 22864983

[119] Aydinlik S Erkisa M Cevatemre B Sarimahmut M Dere E Ari F. Enhanced cytotoxic activity of doxorubicin through the inhibition of autophagy in triple negative breast cancer cell line. Biochim Biophys Acta (BBA) - Gen Subj. (2017) 1861:49–57. doi: 10.1016/j.bbagen.2016.11.013, PMID: 27842219

[120] Guo B Tam A Santi SA Parissenti AM. Role of autophagy and lysosomal drug sequestration in acquired resistance to doxorubicin in MCF-7 cells. BMC Cancer. (2016) 16:762. doi: 10.1186/s12885-016-2790-3, PMID: 27687594 PMC5043608

[121] Liu Z Shi A Song D Han B Zhang Z Ma L. Resistin confers resistance to doxorubicin-induced apoptosis in human breast cancer cells through autophagy induction. Am J Cancer Res. (2017) 7:574–83., PMID: 28401013 PMC5385645

[122] Zhao D Yuan H Yi F Meng C Zhu Q. Autophagy prevents doxorubicin−induced apoptosis in osteosarcoma. Mol Med Rep. (2014) 9:1975–81. doi: 10.3892/mmr.2014.2055, PMID: 24639013

[123] Xu XD Zhao Y Zhang M He RZ Shi XH Guo XJ. Inhibition of autophagy by deguelin sensitizes pancreatic cancer cells to doxorubicin. Int J Mol Sci. (2017) 18:370. doi: 10.3390/ijms18020370, PMID: 28208617 PMC5343905

[124] Li J Zhou W Mao Q Gao D Xiong L Hu X. HMGB1 promotes resistance to doxorubicin in human hepatocellular carcinoma cells by inducing autophagy via the AMPK/mTOR signaling pathway. Front Oncol. (2021) 11:739145. doi: 10.3389/fonc.2021.739145, PMID: 34778055 PMC8578906

[125] Matsunaga T Kawabata S Yanagihara Y Kezuka C Kato M Morikawa Y. Pathophysiological roles of autophagy and aldo-keto reductases in development of doxorubicin resistance in gastrointestinal cancer cells. Chemico-Biological Interact. (2019) 314:108839. doi: 10.1016/j.cbi.2019.108839, PMID: 31563593

[126] Zhu H Jiang C-W Zhang W-L Yang Z-Y Sun G. Targeting oncogenic MAGEA6 sensitizes triple negative breast cancer to doxorubicin through its autophagy and ferroptosis by stabling AMPKα1. Cell Death Discov. (2024) 10:430. doi: 10.1038/s41420-024-02196-9, PMID: 39370446 PMC11456603

[127] Amani N Shaki F Shokrzadeh M. Contribution of autophagy in acquired drug resistance of human breast cancer cells MCF7 to doxorubicin. Appl In Vitro Toxicol. (2019) 5:173–9. doi: 10.1089/aivt.2019.0007

[128] Shi Y Ye Z Lu G Yang N Zhang J Wang L. Cholesterol-enriched membrane micro-domain deficiency induces doxorubicin resistance via promoting autophagy in breast cancer. Mol Ther - Oncolytics. (2021) 23:311–29. doi: 10.1016/j.omto.2021.10.005, PMID: 34786475 PMC8573103

[129] Fei Y Yan X Liang M Zhou S Xu D Li L. Lysosomal gene ATP6AP1 promotes doxorubicin resistance via up-regulating autophagic flux in breast cancer. Cancer Cell Int. (2024) 24:394. doi: 10.1186/s12935-024-03579-9, PMID: 39627767 PMC11616228

[130] Tan Q Wang H Hu Y Hu M Li X A. Src/STAT3-dependent heme oxygenase-1 induction mediates chemoresistance of breast cancer cells to doxorubicin by promoting autophagy. Cancer Sci. (2015) 106:1023–32. doi: 10.1111/cas.12712, PMID: 26041409 PMC4556392

[131] Liao Y-X Lv J-Y Zhou Z-F Xu T-Y Yang D Gao Q-M. CXCR4 blockade sensitizes osteosarcoma to doxorubicin by inducing autophagic cell death via PI3K−Akt−mTOR pathway inhibition. Int J Oncol. (2021) 59:49. doi: 10.3892/ijo.2021.5229, PMID: 34080667 PMC8208619

[132] Thomas M Davis T Nell T Sishi B Engelbrecht AM. Amino acid starvation sensitizes resistant breast cancer to doxorubicin-induced cell death. Front Cell Dev Biol. (2020) 8:565915. doi: 10.3389/fcell.2020.565915, PMID: 33178685 PMC7593593

[133] Yang M-Y Lin P-M Liu Y-C Hsiao H-H Yang W-C Hsu J-F. Induction of cellular senescence by doxorubicin is associated with upregulated miR-375 and induction of autophagy in K562 cells. PLoS One. (2012) 7:e37205. doi: 10.1371/journal.pone.0037205, PMID: 22606351 PMC3350486

[134] Natu A Pedgaonkar A Gupta S. Mitochondrial dysfunction and chromatin changes with autophagy-mediated survival in doxorubicin resistant cancer cell lines. Biochem Biophys Res Commun. (2023) 648:1–10. doi: 10.1016/j.bbrc.2023.01.081, PMID: 36724554

[135] Liao C-C Long Y Tsai M-L Lin C-Y Hsu K-W Lee C-H. G-cleave LC3B biosensor: monitoring autophagy and assessing resveratrol’s synergistic impact on doxorubicin-induced apoptosis in breast cancer cells. Breast Cancer Res. (2024) 26:190. doi: 10.1186/s13058-024-01951-1, PMID: 39736723 PMC11687128

[136] Cosan D Soyocak A Tekedereli I Gacar G Karaoz E Ozpolat B. Abstract 5109: Doxorubicin-induced autophagy functions as a pro-survival pathway in breast cancer cells. Cancer Res. (2010) 70:5109–9. doi: 10.1158/1538-7445.AM10-5109

[137] Hua F Xiao Y-Y Qu X-H Li S-S Zhang K Zhou C. Baicalein sensitizes triple negative breast cancer MDA-MB-231 cells to doxorubicin via autophagy-mediated down-regulation of CDK1. Mol Cell Biochem. (2023) 478:1519–31. doi: 10.1007/s11010-022-04597-9, PMID: 36413334

[138] Zhou J Li G Zheng Y Shen H-M Hu X Ming Q-L. A novel autophagy/mitophagy inhibitor liensinine sensitizes breast cancer cells to chemotherapy through DNM1L-mediated mitochondrial fission. Autophagy. (2015) 11:1259–79. doi: 10.1080/15548627.2015.1056970, PMID: 26114658 PMC4590597

[139] Zhong J Sun P Xu N Liao M Xu C Ding Y. Canagliflozin inhibits p-gp function and early autophagy and improves the sensitivity to the antitumor effect of doxorubicin. Biochem Pharmacol. (2020) 175:113856. doi: 10.1016/j.bcp.2020.113856, PMID: 32061772

[140] Coronel-Hernández J Salgado-García R Cantú-DeLeón D Jacobo-Herrera N Millan-Catalan O Delgado-Waldo I. Combination of metformin, sodium oxamate and doxorubicin induces apoptosis and autophagy in colorectal cancer cells via downregulation HIF-1α. Front Oncol. (2021) 11:594200. doi: 10.3389/fonc.2021.594200, PMID: 34123772 PMC8187873

[141] Yang F Wang F Liu Y Wang S Li X Huang Y. Sulforaphane induces autophagy by inhibition of HDAC6-mediated PTEN activation in triple negative breast cancer cells. Life Sci. (2018) 213:149–57. doi: 10.1016/j.lfs.2018.10.034, PMID: 30352240

[142] Yin W Pham CV Wang T AlShamaileh H Chowdhury R Patel S. Inhibition of autophagy promotes the elimination of liver cancer stem cells by CD133 aptamer-targeted delivery of doxorubicin. Biomolecules. (2022) 12:1623. doi: 10.3390/biom12111623, PMID: 36358973 PMC9687680

[143] Hseu Y-C Lin R-W Shen Y-C Lin K-Y Liao J-W Thiyagarajan V. Flavokawain B and doxorubicin work synergistically to impede the propagation of gastric cancer cells via ROS-mediated apoptosis and autophagy pathways. Cancers. (2020) 12:2475. doi: 10.3390/cancers12092475, PMID: 32882870 PMC7564097

[144] Lin L-T Uen W-C Choong C-Y Shi Y-C Lee B-H Tai C-J. Paris polyphylla inhibits colorectal cancer cells via inducing autophagy and enhancing the efficacy of chemotherapeutic drug doxorubicin. Molecules. (2019) 24:2102. doi: 10.3390/molecules24112102, PMID: 31163662 PMC6600962

[145] Li J. Chidamide enhances cytotoxicity of doxorubicin by promoting autophagy and apoptosis in breast cancer. BMC Cancer. (2023) 23:353. doi: 10.1186/s12885-023-10774-w, PMID: 37069549 PMC10111806

[146] Chen H Zhao C He R Zhou M Liu Y Guo X. Danthron suppresses autophagy and sensitizes pancreatic cancer cells to doxorubicin. Toxicol Vitro. (2019) 54:345–53. doi: 10.1016/j.tiv.2018.10.019, PMID: 30389604

[147] Fong MY Jin S Rane M Singh RK Gupta R Kakar SS. Withaferin A synergizes the therapeutic effect of doxorubicin through ROS-mediated autophagy in ovarian cancer. PLoS One. (2012) 7:e42265. doi: 10.1371/journal.pone.0042265, PMID: 22860102 PMC3408484

[148] Wei T Xiaojun X Peilong C. Magnoflorine improves sensitivity to doxorubicin (DOX) of breast cancer cells via inducing apoptosis and autophagy through AKT/mTOR and p38 signaling pathways. Biomedicine Pharmacotherapy. (2020) 121:109139. doi: 10.1016/j.biopha.2019.109139, PMID: 31707337

[149] Khan I Bahuguna A Bhardwaj M PalKhaket T Kang SC. Carvacrol nanoemulsion evokes cell cycle arrest, apoptosis induction and autophagy inhibition in doxorubicin resistant-A549 cell line. Artif Cells Nanomedicine Biotechnol. (2018) 46:664–75. doi: 10.1080/21691401.2018.1434187, PMID: 29405784

[150] Friedhuber AM Chandolu V Manchun S Donkor O Sriamornsak P Dass CR. Nucleotropic doxorubicin nanoparticles decrease cancer cell viability, destroy mitochondria, induce autophagy and enhance tumour necrosis. J Pharm Pharmacol. (2015) 67:68–77. doi: 10.1111/jphp.12322, PMID: 25208603

[151] Kulkarni-Dwivedi N Patel PR Shravage BV Umrani RD Paknikar KM Jadhav SH. Hyperthermia and doxorubicin release by fol-LSMO nanoparticles induce apoptosis and autophagy in breast cancer cells. Nanomedicine. (2022) 17:1929–49. doi: 10.2217/nnm-2022-0171, PMID: 36645007

[152] Gao M Xu Y Qiu L. Sensitization of multidrug-resistant Malignant cells by liposomes co-encapsulating doxorubicin and chloroquine through autophagic inhibition. J Liposome Res. (2017) 27:151–60. doi: 10.1080/08982104.2016.1185731, PMID: 27250110

[153] Yang Q Zhang W Lu S-Y Cai X Chen C Zhang Q. Biodegradable doxorubicin-loaded ferric phosphate nanosheets for sp*ecific tumor elimination through autophagy inhibition-enhanced apoptosis/ferroptosis pathway* . Chem Eng J. (2023) 454:140455. doi: 10.1016/j.cej.2022.140455

[154] Zhang H Xue Q Zhou Z He N Li S Zhao C. Co-delivery of doxorubicin and hydroxychloroquine via chitosan/alginate nanoparticles for blocking autophagy and enhancing chemotherapy in breast cancer therapy. Front Pharmacol. (2023) 14:1176232. doi: 10.3389/fphar.2023.1176232, PMID: 37229260 PMC10203398

[155] Wang J Qiu L. Drug-induced self-assembled nanovesicles for doxorubicin resistance reversal via autophagy inhibition and delivery synchronism. Theranostics. (2022) 12:3977–94. doi: 10.7150/thno.70852, PMID: 35664062 PMC9131275

[156] Wang F-Z Xing L Tang Z-h Lu J-J Cui P-F Qiao J-B. Codelivery of doxorubicin and shAkt1 by poly(ethylenimine)–glycyrrhetinic acid nanoparticles to induce autophagy-mediated liver cancer combination therapy. Mol Pharmaceutics. (2016) 13:1298–307. doi: 10.1021/acs.molpharmaceut.5b00879, PMID: 26894988

[157] Jin W Yang T Jia J Jia J Zhou X. Enhanced sensitivity of A549 cells to doxorubicin with WS2 and WSe2 nanosheets via the induction of autophagy. Int J Mol Sci. (2024) 25:1164. doi: 10.3390/ijms25021164, PMID: 38256235 PMC10816038

[158] Liang L Fu J Wang S Cen H Zhang L Mandukhail SR. MiR-142-3p enhances chemosensitivity of breast cancer cells and inhibits autophagy by targeting HMGB1. Acta Pharm Sin B. (2020) 10:1036–46. doi: 10.1016/j.apsb.2019.11.009, PMID: 32642410 PMC7332808

[159] Bao LJ Jaramillo MC Zhang ZB Zheng YX Yao M Zhang DD. Nrf2 induces cisplatin resistance through activation of autophagy in ovarian carcinoma. Int J Clin Exp Pathol. (2014) 7:1502–13., PMID: 24817946 PMC4014230

[160] Chittaranjan S Bortnik S Dragowska WH Xu J Abeysundara N Leung A. Autophagy inhibition augments the anticancer effects of epirubicin treatment in anthracycline-sensitive and -resistant triple-negative breast cancer. Clin Cancer Res. (2014) 20:3159–73. doi: 10.1158/1078-0432.CCR-13-2060, PMID: 24721646

[161] Wang W Chen D Zhu K. SOX2OT variant 7 contributes to the synergistic interaction between EGCG and Doxorubicin to kill osteosarcoma via autophagy and stemness inhibition. J Exp Clin Cancer Res. (2018) 37:37. doi: 10.1186/s13046-018-0689-3, PMID: 29475441 PMC6389193

[162] Chiu W-J Lin C-S Lin S-R Chen T-H Wu C-J Busa P. Diterpene promptly executes a non-canonical autophagic cell death in doxorubicin-resistant lung cancer. Biomedicine Pharmacotherapy. (2022) 153:113443. doi: 10.1016/j.biopha.2022.113443, PMID: 36076558

[163] Zhang P Liu X Li H Chen Z Yao X Jin J. TRPC5-induced autophagy promotes drug resistance in breast carcinoma via CaMKKβ/AMPKα/mTOR pathway. Sci Rep. (2017) 7:3158. doi: 10.1038/s41598-017-03230-w, PMID: 28600513 PMC5466655

[164] Wang Y Yi Y Yao J Wan H Yu M Ge L. Isoginkgetin synergizes with doxorubicin for robust co-delivery to induce autophagic cell death in hepatocellular carcinoma. Acta Biomaterialia. (2022) 153:518–28. doi: 10.1016/j.actbio.2022.09.035, PMID: 36152910

[165] Bosch-Barrera J Estévez-García P Martín-Martorell P Sabatier R Nadal E Sais E. ENDOLUNG trial, part II. A phase II study of the Akt/mTOR inhibitor and autophagy inducer ibrilatazar (ABTL0812) in combination with paclitaxel/carboplatin in patients with squamous non-small cell lung cancer. Lung Cancer. (2025) 201:108105. doi: 10.1016/j.lungcan.2025.108105, PMID: 39983444

[166] Ueno T Masuda N Kamigaki S Morimoto T Saji S Imoto S. Differential involvement of autophagy and apoptosis in response to chemoendocrine and endocrine therapy in breast cancer: JBCRG-07TR. Int J Mol Sci. (2019) 20. doi: 10.3390/ijms20040984, PMID: 30813476 PMC6412499

[167] Zeh HJ Bahary N Boone BA Singhi AD Miller-Ocuin JL Normolle DP. A randomized phase II preoperative study of autophagy inhibition with high-dose hydroxychloroquine and gemcitabine/nab-paclitaxel in pancreatic cancer patients. Clin Cancer Res. (2020) 26:3126–34. doi: 10.1158/1078-0432.CCR-19-4042, PMID: 32156749 PMC8086597

[168] Berger MD Yamauchi S Cao S Hanna DL Sunakawa Y Schirripa M. Autophagy-related polymorphisms predict hypertension in patients with metastatic colorectal cancer treated with FOLFIRI and bevacizumab: Results from TRIBE and FIRE-3 trials. Eur J Cancer. (2017) 77:13–20. doi: 10.1016/j.ejca.2017.02.020, PMID: 28347919 PMC7497847

[169] Mahalingam D Mita M Sarantopoulos J Wood L Amaravadi RK Davis LE. Combined autophagy and HDAC inhibition: a phase I safety, tolerability, pharmacokinetic, and pharmacodynamic analysis of hydroxychloroquine in combination with the HDAC inhibitor vorinostat in patients with advanced solid tumors. Autophagy. (2014) 10:1403–14. doi: 10.4161/auto.29231, PMID: 24991835 PMC4203517

[170] Gong C Lin Q Qin T Zeng Y Xu F Yang Y. Targeting autophagy plus high-dose CDK4/6 inhibitors in advanced HR+HER2- breast cancer: A phase 1b/2 trial. Med. (2025) 6:100559. doi: 10.1016/j.medj.2024.11.012, PMID: 39731909

[171] Rangwala R Chang YC Hu J Algazy KM Evans TL Fecher LA. Combined MTOR and autophagy inhibition: phase I trial of hydroxychloroquine and temsirolimus in patients with advanced solid tumors and melanoma. Autophagy. (2014) 10:1391–402. doi: 10.4161/auto.29119, PMID: 24991838 PMC4203516

[172] Hansen AR Tannock IF Templeton A Chen E Evans A Knox J. Pantoprazole affecting docetaxel resistance pathways via autophagy (PANDORA): phase II trial of high dose pantoprazole (Autophagy inhibitor) with docetaxel in metastatic castration-resistant prostate cancer (mCRPC). Oncologist. (2019) 24:1188–94. doi: 10.1634/theoncologist.2018-0621, PMID: 30952818 PMC6738292

[173] Lopez J Lai-Kwon J Molife R Welsh L Tunariu N Roda D. A Phase 1/2A trial of idroxioleic acid: first-in-class sp*hingolipid regulator and glioma cell autophagy inducer with antitumor activity in refractory glioma* . Br J Cancer. (2023) 129:811–8. doi: 10.1038/s41416-023-02356-1, PMID: 37488446 PMC10449773

[174] Wolpin BM Rubinson DA Wang X Chan JA Cleary JM Enzinger PC. Phase II and pharmacodynamic study of autophagy inhibition using hydroxychloroquine in patients with metastatic pancreatic adenocarcinoma. Oncologist. (2014) 19:637–8. doi: 10.1634/theoncologist.2014-0086, PMID: 24821822 PMC4041680

[175] Parker KH Horn LA Ostrand-Rosenberg S. High-mobility group box protein 1 promotes the survival of myeloid-derived suppressor cells by inducing autophagy. J Leukocyte Biol. (2016) 100:463–70. doi: 10.1189/jlb.3HI0715-305R, PMID: 26864266 PMC4982609

[176] Zhang W Li X Jiang M Ji C Chen G Zhang Q. SOCS3 deficiency-dependent autophagy repression promotes the survival of early-stage myeloid-derived suppressor cells in breast cancer by activating the Wnt/mTOR pathway. J Leukocyte Biol. (2023) 113:445–60. doi: 10.1093/jleuko/qiad020, PMID: 36808484

[177] Wei W-F Zhou H-L Chen P-Y Huang X-L Huang L Liang L-J. Cancer-associated fibroblast-derived PAI-1 promotes lymphatic metastasis via the induction of EndoMT in lymphatic endothelial cells. J Exp Clin Cancer Res. (2023) 42:160. doi: 10.1186/s13046-023-02714-0, PMID: 37415190 PMC10324144

[178] Li X-F Chen D-P Ouyang F-Z Chen M-M Wu Y Kuang D-M. Increased autophagy sustains the survival and pro-tumourigenic effects of neutrophils in human hepatocellular carcinoma. J Hepatol. (2015) 62:131–9. doi: 10.1016/j.jhep.2014.08.023, PMID: 25152203

[179] Janji B Hasmim M Parpal S De Milito A Berchem G Noman MZ. Lighting up the fire in cold tumors to improve cancer immunotherapy by blocking the activity of the autophagy-related protein PIK3C3/VPS34. Autophagy. (2020) 16:2110–1. doi: 10.1080/15548627.2020.1815439, PMID: 32892693 PMC7595609

[180] Mgrditchian T Arakelian T Paggetti J Noman MZ Viry E Moussay E. Targeting autophagy inhibits melanoma growth by enhancing NK cells infiltration in a CCL5-dependent manner. Proc Natl Acad Sci. (2017) 114:E9271–9. doi: 10.1073/pnas.1703921114, PMID: 29078276 PMC5676879

[181] Taraborrelli L Şenbabaoğlu Y Wang L Lim J Blake K Kljavin N. Tumor-intrinsic expression of the autophagy gene Atg16l1 suppresses anti-tumor immunity in colorectal cancer. Nat Commun. (2023) 14:5945. doi: 10.1038/s41467-023-41618-7, PMID: 37741832 PMC10517947

[182] Zhang W Chen L Liu J Chen B Shi H Chen H. Inhibition of autophagy-related protein 7 enhances anti-tumor immune response and improves efficacy of immune checkpoint blockade in microsatellite instability colorectal cancer. J Exp Clin Cancer Res. (2024) 43:114. doi: 10.1186/s13046-024-03023-w, PMID: 38627815 PMC11020677

[183] Jin Y Qiu J Lu X Li G. C-MYC inhibited ferroptosis and promoted immune evasion in ovarian cancer cells through NCOA4 mediated ferritin autophagy. Cells. (2022) 11:4127. doi: 10.3390/cells11244127, PMID: 36552889 PMC9776536

[184] Zhan L Zhang J Wei B Cao Y. Selective autophagy of NLRC5 promotes immune evasion of endometrial cancer. Autophagy. (2022) 18:942–3. doi: 10.1080/15548627.2022.2037119, PMID: 35174769 PMC9037573

[185] Yang D Chen M Sun Y Shi C Wang W Zhao W. Microneedle-assisted vaccination combined with autophagy regulation for antitumor immunotherapy. J Controlled Release. (2023) 357:641–54. doi: 10.1016/j.jconrel.2023.04.031, PMID: 37084892

[186] Xiao H Li X Li B Yang S Qin J Han S. Nanodrug inducing autophagy inhibition and mitochondria dysfunction for potentiating tumor photo-immunotherapy. Small. (2023) 19:2300280. doi: 10.1002/smll.202300280, PMID: 37060227

[187] Xia C Li M Ran G Wang X Lu Z Li T. Redox-responsive nanoassembly restrained myeloid-derived suppressor cells recruitment through autophagy-involved lactate dehydrogenase A silencing for enhanced cancer immunochemotherapy. J Controlled Release. (2021) 335:557–74. doi: 10.1016/j.jconrel.2021.05.034, PMID: 34051289

[188] Wang Y Lin Y-X Wang J Qiao S-L Liu Y-Y Dong W-Q. In situ manipulation of dendritic cells by an autophagy-regulative nanoactivator enables effective cancer immunotherapy. ACS Nano. (2019) 13:7568–77. doi: 10.1021/acsnano.9b00143, PMID: 31260255

[189] Li M Zhao D Yan J Fu X Li F Liu G. A redox-triggered autophagy-induced nanoplatform with PD-L1 inhibition for enhancing combined chemo-immunotherapy. ACS Nano. (2024) 18:12870–84. doi: 10.1021/acsnano.4c00227, PMID: 38727063

[190] Wang X Li M Ren K Xia C Li J Yu Q. On-demand autophagy cascade amplification nanoparticles precisely enhanced oxaliplatin-induced cancer immunotherapy. Advanced Materials. (2020) 32:2002160. doi: 10.1002/adma.202002160, PMID: 32596861

[191] Long X Wang H Yan J Li Y Dong X Tian S. Tailor-made autophagy cascade amplification polymeric nanoparticles for enhanced tumor immunotherapy. Small. (2023) 19:2207898. doi: 10.1002/smll.202207898, PMID: 36932938

[192] Zhang L Jia Y Yang J Zhang L Hou S Niu X. Efficient immunotherapy of drug-free layered double hydroxide nanoparticles via neutralizing excess acid and blocking tumor cell autophagy. ACS Nano. (2022) 16:12036–48. doi: 10.1021/acsnano.2c02183, PMID: 35881002

[193] Ouyang Z Gao Y Shen S Jia B Yu H Wang H. A minimalist dendrimer nanodrug for autophagy inhibition-amplified tumor photothermo-immunotherapy. Nano Today. (2023) 51:101936. doi: 10.1016/j.nantod.2023.101936

[194] Zhang S Xie F Li K Zhang H Yin Y Yu Y. Gold nanoparticle-directed autophagy intervention for antitumor immunotherapy via inhibiting tumor-associated macrophage M2 polarization. Acta Pharm Sin B. (2022) 12:3124–38. doi: 10.1016/j.apsb.2022.02.008, PMID: 35865102 PMC9293675

[195] Gao C Kwong CHT Wang Q Kam H Xie B Lee SM-Y. Conjugation of macrophage-mimetic microalgae and liposome for antitumor sonodynamic immunotherapy via hypoxia alleviation and autophagy inhibition. ACS Nano. (2023) 17:4034–49. doi: 10.1021/acsnano.3c00041, PMID: 36739531

